# Development and validation of an improved algorithm for overlaying flexible molecules

**DOI:** 10.1007/s10822-012-9573-y

**Published:** 2012-04-27

**Authors:** Robin Taylor, Jason C. Cole, David A. Cosgrove, Eleanor J. Gardiner, Valerie J. Gillet, Oliver Korb

**Affiliations:** 1 Taylor Cheminformatics Software, 54 Sherfield Avenue, Rickmansworth, Hertfordshire, WD3 1NL UK; 2 Cambridge Crystallographic Data Centre, 12 Union Road, Cambridge, CB2 1EZ UK; 3 AstraZeneca Pharmaceuticals, Mereside, Alderley Park, Macclesfield, SK10 4TG UK; 4 Information School, University of Sheffield, Regent Court, 211 Portobello Street, Sheffield, S1 4DP UK

**Keywords:** Alignment, Overlay, Pharmacophore

## Abstract

A program for overlaying multiple flexible molecules has been developed. Candidate overlays are generated by a novel fingerprint algorithm, scored on three objective functions (union volume, hydrogen-bond match, and hydrophobic match), and ranked by constrained Pareto ranking. A diverse subset of the best ranked solutions is chosen using an overlay-dissimilarity metric. If necessary, the solutions can be optimised. A multi-objective genetic algorithm can be used to find additional overlays with a given mapping of chemical features but different ligand conformations. The fingerprint algorithm may also be used to produce constrained overlays, in which user-specified chemical groups are forced to be superimposed. The program has been tested on several sets of ligands, for each of which the true overlay is known from protein–ligand crystal structures. Both objective and subjective success criteria indicate that good results are obtained on the majority of these sets.

**Electronic supplementary material:**

The online version of this article (doi:10.1007/s10822-012-9573-y) contains supplementary material, which is available to authorized users.

## Introduction

Ligand-based drug design techniques such as pharmacophore analysis [1] and 3D quantitative structure–activity relationships (3D QSAR) [2] are widely used. They usually require the alignment of a set of ligands known to bind to the same protein. When the protein structure is unknown, the likelihood that a given overlay is correct can be judged by the extent to which it places similar groups from different ligands near to one another, and on the energies of the ligand conformations. If the ligands are flexible, there can be an enormous number of ways in which they could be overlaid. The problem is therefore challenging. New molecular-overlay algorithms continue to be published [3–16], suggesting that the state of the art is not considered satisfactory.

In the absence of the protein structure, the molecular-overlay problem is under-determined. Except in trivial cases, it is therefore unreasonable to suppose that the correct solution can be identified unambiguously. A more realistic aspiration is to produce a small number of significantly different but credible alignments, one of which is close to the truth. With this in mind, we have previously investigated the use of a multiple-objective genetic algorithm (MOGA) for molecular alignment and pharmacophore elucidation [17–19]. Our method is designed to produce several overlays of a set of ligands using Pareto ranking [20]. Each represents a different trade-off between the various objective functions measuring overlay quality, such as strain energy, volume, and matching of hydrogen-bond features. The generation of multiple diverse overlays produces a range of pharmacophore hypotheses to test.

While results were promising, we were aware of several opportunities for improvement. For example, some types of hydrophobic features were not properly represented; the scoring protocol sometimes underestimated the degree of hydrogen-bond matching; clique detection was used to set up starting overlays for optimisation by the MOGA, but other approaches seemed worth investigating. We also wished to make analysis of the results easier: if a molecular-overlay program produces many possible solutions, it can be time consuming to sift through the output. We therefore wanted good measures of overlay similarity that would enable solutions to be clustered, or mapped in low-dimensional space. Finally, we decided to test the revised algorithm on several new sets of ligands, including diverse sets, typical of those used as input for pharmacophore elucidation, and sets of relatively close homologues, such as are used in 3D QSAR.

The outcome of our new work has been to change the algorithm appreciably. A novel method has been developed for generating promising overlays using bit-string manipulations. The resulting overlays are scored using new objective functions, Pareto-ranked, and a diverse subset of the best-ranked solutions chosen using an overlay-dissimilarity measure. Overlays can be refined, subjected to a new process we call “overlay multiplication”, and mapped using multidimensional scaling. The new algorithm has been tested on 10 sets of ligands taken from protein–ligand crystal structures in the Protein Data Bank (PDB) [21].

## Methods

### Organisation and overview

This section is organised as follows. We begin by defining key terms and summarising the molecular input required by the program. We then describe how chemical features such as hydrogen-bonding and hydrophobic groups are identified and represented. This is followed by a description of several scoring functions used to assess the quality of solution overlays.

We then describe the search algorithm, the first step of which is *overlay generation*. This is a fingerprint technique which generates several thousand possible overlays using bit-string manipulations. The second step, *overlay filtering*, uses the scoring functions referred to above, together with overlay similarity calculations, to identify a diverse subset of the best of the solutions that have been generated. Optionally, some or all of these may be subjected to *overlay* *refinement*—an optimisation process to bring approximately aligned groups into closer alignment. Finally, a procedure called *overlay multiplication* may be applied to solutions of particular interest. This explores the geometric variability of specific pharmacophore hypotheses, using a MOGA to determine whether a particular superposition of ligand chemical features can be achieved with more than one set of ligand conformations. The overlay generation and filtering steps are critical: if they fail to produce good overlays, it is unlikely that refinement or multiplication will rectify the problem. Conversely, overlays from the filtering step may be good enough that no refinement or multiplication is necessary.

The section ends with a description of how overlay similarity can be quantified, and describes analytical techniques for helping users understand the relationships between different overlays.

### Nomenclature

The molecules to be overlaid are divided into *features* such as hydrogen-bond donors and acceptors, and hydrophobic groups (*hydrophobes*). Each feature is represented by one or more *fitting points* placed at strategic positions (for example, on a donor atom or at the centroid of a hydrophobe). A cluster of fitting points in an overlay, all representing the same type of feature and each from a different ligand, constitutes a *pharmacophore point*. If every ligand contributes, it is a *full pharmacophore point*; otherwise it is a *partial pharmacophore point*. The complete collection of pharmacophore points in an overlay (optionally rejecting partial points involving less than a specified number of ligands) is the *pharmacophore* *hypothesis* (or simply *pharmacophore*) suggested by that overlay. The composition of the pharmacophore (that is, the ligand features that contribute to the pharmacophore points) is the *feature mapping*.

### Ligand preparation

The ligands must be built in the protonation states they are expected to adopt at the protein binding site, as these are not altered during overlaying. While this is a weakness in the program, the numbers of ligands being overlaid will usually be small enough to allow users to assign protonation states manually. Indeed, given knowledge from in-house chemistry, users may often be better placed to decide on difficult tautomeric issues than an algorithm. A set of low-energy conformers must be calculated for each ligand. We have used OMEGA [22] but other conformer generators should also be suitable.

### Feature definition; fitting-point placement

Two types of hydrophobic features are defined, *directional* and *non*-*directional*. The former are groups that are more likely to form hydrophobic interactions in some directions than others, such as aromatic rings [23] and amide linkages. It may seem odd to define amide as a hydrophobe, but inspection of protein–ligand crystal structures (for example, using the IsoStar system [24]) shows that this group tends to form hydrophobic interactions perpendicular to the amide plane, although interactions in the plane are invariably hydrogen bonds. Non-directional hydrophobes are groups that are equally hydrophobic in all directions, such as alkyl chains. Hydrophobes are represented by a fitting point at the centroid. Optionally, two further fitting points may be used for directional hydrophobes, placed on the normal to the least-squares mean plane, one on each side, at 1 Å from the centroid.

The algorithm for defining hydrophobic features is similar to that used by others [25]. All rings of size ≤7 are classed as hydrophobes (directional if at least three of the ring atoms are delocalisable, otherwise non-directional). Groups such as t-butyl and –CF_3_ are considered non-directional hydrophobes. Amide, C=C, C=N and N=N linkages are classed as directional hydrophobes. Other hydrophobic portions of the molecule (acyclic chains, rings of size >7) are divided into segments of up to four atoms, each segment constituting a separate non-directional hydrophobe. Segments of only three or two atoms are chosen if it leads to more uniform placement of fitting points.

All other feature types are customisable, being defined by SMARTS (Smiles arbitrary target specification) strings [26]. Any number of feature types may be defined, such as donors, acceptors, metal coordinators, and positive and negative centres (in this work, we have only used donors and acceptors, the latter serving as a surrogate for metal coordinators). It is necessary to provide a list of SMARTS strings defining the substructures that belong to each feature type. SMARTS strings defining donors or acceptors must be accompanied by two additional data items. One defines the strength of the hydrogen bonds formed by the group, categorised as strong (only used for ionised groups), medium, or weak (thiourea sulfur acceptors and thiol and C–H donors). The second data item specifies the preferred geometry of the hydrogen bonding group. For example, two-coordinate sp^2^ nitrogen is defined as a trigonal acceptor (preferentially hydrogen bonds along its sp^2^ lone-pair direction).

CH groups are only classified as donors if they are in particularly electron withdrawing environments (for example, the 2 position of pyrimidine). Phenyl CH groups are not considered donors. This can make it difficult for the algorithm to reproduce certain unusual overlays. In Factor Xa complexes, for instance, Asp189 often forms strong hydrogen bonds to ligand groups such as amidinium, but it can also interact with phenyl CH groups (for example, see PDB complexes 1lpz and 1iqm). The algorithm, however, will tend not to overlay amidinium NH on phenyl CH.

The location of donor and acceptor fitting points is customisable but in practice we always place them on the donor and acceptor atoms rather than on hydrogen and lone-pair positions, or at the inferred positions of the complementary hydrogen-bonding atoms on the protein. Our choice may make it more difficult to find overlays in which two ligand atoms can hydrogen bond to the same protein atom even though they are not close to each other in the overlay (for example, because they donate to different lone pairs of the same protein carbonyl oxygen). However, this situation occurs rather infrequently (based on an analysis of our test-set complexes) and fitting points at hydrogen, lone pair or inferred protein-atom positions make the search space larger (for example, may require hydrogen-atom torsions to be varied). Also, points lying outside the molecular envelope (that is, at inferred protein-atom positions) tend to have unduly large leverage during overlay generation. Atoms that are both donors and acceptors (notably hydroxyl oxygens) have both a donor and an acceptor point placed on them.

It is possible to exclude particular atoms from feature assignment. For example, the hydrogen-bonding atoms of a ligand solubilising group could be excluded, meaning that no donor or acceptor fitting points will be placed on them. Conversely, special feature types can be defined to contain sets of hand-picked atoms, rather than atoms matching SMARTS strings.

### Scoring functions

Up to five scoring functions are used to quantify overlay quality.

#### Volume score

This is the union volume of all ligands, V, calculated by placing a grid over the overlay and counting the points within the overlay envelope. Small V scores are considered desirable, since ligands need to bind in a cavity of limited size. A grid size of 0.5 Å is used by default. Tests on neprilysin ligand overlays showed that volumes calculated with this grid size may be in error by up to about 0.5 %, which is adequate for our purposes.

#### Hydrogen bond score

Leader-style cluster analysis [27, 28] is used to find clusters of donor and acceptor atoms, each cluster containing only donors or only acceptors, with no more than one atom from any given ligand. A cluster need not include an atom from every ligand. The algorithm works by setting up a “nearest neighbour list” (NNL) for each donor and acceptor (X) in the overlay. For a given X, the NNL contains X itself and the closest donor (or acceptor) to X in each of the other ligands, provided that it is within 1.5 Å of X. NNLs therefore vary in length; for example, if a given X is >1.5 Å away from all donors (or acceptors) in all other ligands, its NNL will only contain X itself. The longest NNL is chosen as the first cluster. All members of this cluster are then removed from the remaining NNLs. The longest of the remaining NNLs is chosen as the second cluster, and so on (if NNLs tie on length, the one with the smaller mean square distance between its members is chosen).

**Fig. 1:**
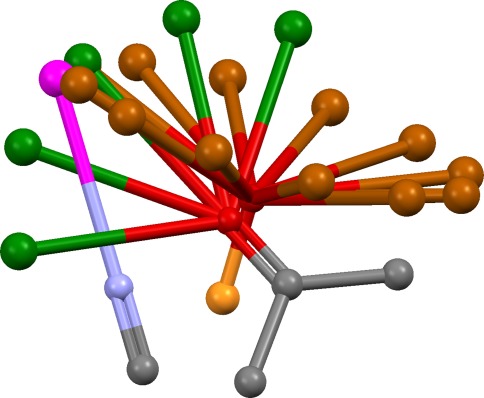
Example overlay of nitrile, carbonyl and phosphate acceptors. Each acceptor is shown with virtual points representing possible positions of the protein donor (*magenta*: nitrile; *green*: carbonyl; *brown*: phosphate). A direction in which all three acceptors can hydrogen bond is indicated by the cluster of virtual points, one from each acceptor, at the *top left* of the figure

The best consensus hydrogen-bonding direction for the atoms in each cluster is then determined. Consider, for example, an acceptor cluster containing phosphate oxygen, carbonyl oxygen and nitrile nitrogen. For each acceptor, “virtual points” are placed to represent the positions at which the complementary protein donor might lie. These are evenly spaced around the base of a cone for phosphate oxygen; in the sp^2^ lone-pair directions and at intermediate positions for carbonyl oxygen; and on the sp axis for nitrile nitrogen. The largest cluster of virtual points is found, using the same clustering method as above. This represents the best consensus direction for hydrogen bonding (Fig. 1). The size of the largest virtual-point cluster might be less than the size of the parent cluster of donor or acceptor atoms, indicating that they cannot all hydrogen-bond in the same direction. Steric accessibility is assessed by placing points on the line between the centroid of the donor or acceptor atoms and the centroid of the chosen virtual-point cluster. Each point is examined to determine whether it falls within the hydrophobic envelope of the overlay and an occlusion factor, X, is calculated which varies from 1 if there is a clear line of sight to 0.1 if the points are highly occluded.

In the protein–ligand structures of our test set, there is no example of a protein atom forming hydrogen bonds to a strong donor or acceptor on one ligand but a weak donor or acceptor on another (using our definitions of strong and weak). Hydrogen bonding is always to ligand groups of the same strength or (less commonly) to a mixture of strong and medium, or medium and weak. To reflect this, the similarity of the donor or acceptor atoms in each cluster is estimated by a factor S = (m/n)^2^, where n is the actual number of atoms in the cluster and m is an “effective” number, set equal to n if all atoms in the cluster have the same strength, but to lower values otherwise.

The hydrogen-bond score (larger values better) is:

1 $$ {\text{HB}} = \Upsigma \left\{ {{\text{S}}_{\text{p}} {\text{X}}_{\text{p}} \left[ {{\text{A}}_{\text{p}}^{2} {\text{f}}({\text{a}}_{\text{p}} ) + {\text{V}}_{\text{p}}^{2} {\text{g}}({\text{v}}_{\text{p}} )} \right]} \right\} $$

Summation is over the donor and acceptor atom clusters (if a set of hydroxyl groups contributes to both donor and acceptor clusters, the contribution of the less well-scoring cluster is ignored). S_p_ and X_p_ are the similarity and occlusion factors for cluster p; A_p_ is the number of atoms in the cluster; V_p_ is the number of virtual points in the largest virtual-point cluster (in the event of a tie, the score is calculated for each in turn and the highest value taken); a_p_ is the mean square distance of the atoms from their centroid; v_p_ is the corresponding quantity for the virtual-points. f(a_p_) is a weighting function which falls linearly from 1.0 to 0.3 as a_p_ increases from 0.15 to 0.75 Å^2^, taking constant values of 1.0 and 0.3, respectively, below and above these distances; g(v_p_) is similar but falls between 1.0 and 0.3 as v_p_ varies from 0.5 to 1.5 Å^2^. The effect is to reward tight clusters.

#### Hydrophobic score

Leader cluster analysis (see above) is used to find clusters of directional hydrophobes. A cluster may contain no more than one hydrophobe from each ligand and need not contain a hydrophobe from every ligand. Inter-planar angles are calculated between all pairs of hydrophobes in each cluster. The score (larger values better) is:

2 $$ {\text{HY}} = \Upsigma \left\{ {{\text{N}}_{\text{p}}^{2} [{\text{f}}({\text{n}}_{\text{p}} ) + {\text{g}}({\text{c}}_{\text{p}} )]} \right\} $$

Summation is over the clusters. N_p_ is the number of hydrophobes in cluster p; n_p_ is the mean-square distance of the centroids of the hydrophobes in the cluster from the mean position of these centroids; c_p_ is the average cosine of the inter-planar angles. f(n_p_) is a weighting function which falls linearly from 1.0 to 0.0 as n_p_ increases from 0.0 to 1.25 Å^2^, remaining constant at zero thereafter, g(c_p_) is similar in form but falls from 2.0 to 0.0 as c_p_ decreases from 1.0 to 0.8. Hence, more weight is placed on the hydrophobes being coplanar than on their centroids being coincident.

#### Energy score

This is the sum of the strain energies of the overlaid ligands, E, calculated from the torsional and van der Waals (vdw) terms of the Tripos force field [29]. Only repulsive atom–atom interactions are included in the vdw sum, to avoid attractive interactions artificially favouring folded conformations. As bond angles are not allowed to relax, all vdw radii are reduced to 0.85 times their published values; a similar approach has been used previously by others [30]. Also, the worst atom–atom clash is ignored provided its energy is <150 kcal/mol, making the function more forgiving when a conformation has a single bad atom–atom clash that could probably be relieved if bond angles were allowed to vary.

#### Customised-feature score

This is employed when the user has defined customised features (feature types other than hydrophobes, donors or acceptors). Clusters of customised-feature centroids are found, each cluster containing only one type of customised feature and no more than one centroid from each ligand. The score (larger values better) is:

3 $$ {\text{CF}} = \Upsigma \left\{ {{\text{ N}}_{\text{p}}^{2} {\text{f}}({\text{n}}_{\text{p}} )} \right\} $$

Summation is over the clusters. N_p_ is the number of customised-feature centroids in cluster p; n_p_ is the mean-square distance between the customised-feature centroids and their overall centroid; f(n_p_) is a weighting function which falls linearly from 1.0 to 0.0 as n_p_ increases from 0.0 to 1.25 Å^2^, remaining constant at zero thereafter.

### Chromosome structure

At some stages of the algorithm, it is convenient to represent overlays not by their atomic coordinates but as a compact representation which we call a chromosome. We use this name because, amongst other uses, chromosomes are used to represent solutions in a MOGA during overlay multiplication. However, they are also used for other purposes unrelated to genetic algorithms: they provide a concise way of storing the large numbers of putative solutions produced by the overlay-generation stage of the algorithm; and they are used for efficient persistent storage of solutions.

A chromosome must fully define the conformation, position and orientation of each ligand. A ligand conformation is defined by: (a) a conformer index, which refers to one of the low-energy conformations supplied by the user; (b) a set of torsion-angle values for the acyclic rotatable bonds. (A file of SMARTS strings is used to indicate which types of acyclic bonds are to be considered rotatable and can also be used to set allowed torsion-angle ranges. For example, we do not rotate methyl groups, and constrain esters to lie within 5° of the trans planar geometry.) The required conformation is generated by setting the molecular geometry to that of the specified conformer and then driving the rotatable bonds to their required torsion settings. The chromosome may contain no torsion data, in which case the indicated conformer is used directly. When torsion angles are supplied, it is still necessary to specify a conformer index in case the ligand contains a flexible ring or invertible nitrogen, in which case different conformers in the input file might have different ring or nitrogen geometries.

The positions and orientations of the ligands are defined by a mapping table which specifies a matching of fitting points. For example, for a three-ligand overlay it might look like:

- ligand A: 1 7 9; ligand B: 4 6 10; ligand C: 2 4 6

This means that ligand B is to be overlaid on ligand A (once they have been set to their specified conformations) by least-squares superposition of its fitting points 4, 6, 10 on 1, 7, 9, respectively, of ligand A. Ligand C is overlaid by least-squares superposition of its fitting points 2, 4, 6 on points a, b, c, where a is the centroid of fitting point 1 (ligand A) and fitting point 4 (ligand B), and so on. The table may contain more than three columns and missing values are allowed. If there are fewer than three columns in the table with no missing values, the algorithm will search for an order in which the ligands can be overlaid. If none can be found, the chromosome is invalid.

The chromosome may also contain three translations and three Euler angles per ligand. If so, the ligand positions are further modified after the mapping-table superpositions by rigid-body rotations about the x, y and z directions, followed by translations. This was implemented to allow ligands to rotate and translate freely during overlay refinement and multiplication.

### Overlay generation

This involves three stages: triplet counting, fingerprint calculation, and fingerprint searching. For simplicity, the procedure will be described assuming that only donor, acceptor and hydrophobe feature types are in use, but extension to more feature types is straightforward.

#### Triplet counting

A triplet is defined as three fitting points from the same conformation of a ligand. Triplets can be classified into types, defined by: (a) the nature of the features that the three fitting points represent (donors, acceptors or hydrophobes); (b) the inter-point distances. By using a set of non-overlapping distance bins, each inter-point distance can be assigned uniquely to one bin, so each triplet can be assigned uniquely to one triplet type. The first step is to find the triplet types that occur most often in the ligand conformations that the user has supplied. All triplets are enumerated and typed. Let L_i_ be the number of ligands in which at least one triplet of type i occurs in at least one conformation. Let P_ij_ be the proportion of conformations of ligand j that contain at least one triplet of type i. Let P_i_ be the average of the P_ij_ over all ligands. Triplet types are sorted in descending order of L_i_ and, in the event of ties, in descending order of P_i_. The position of a triplet type in the sorted list is its rank, starting at 1 for the most common. Let M be the rank of the lowest-ranked triplet type that occurs in all ligands. Overlay generation, as described below, proceeds by iterating over the triplet types from rank 1 to N, where N is the lesser of M and a user-defined value (set to 25 for the validation described below). In each iteration step, overlays are generated by superposition of triplets of the type under consideration in that step. Overlays from all iteration steps are pooled and taken forward to the filtering stage.

All the results discussed below were obtained using triplets derived solely from fitting points placed on acceptors, donors and the centroids of hydrophobic groups. However, we have found that it can sometimes be advantageous to also allow triplets containing fitting points on the normals to directional hydrophobes.

The use of distance bins may lead to a problem. Two triplets that are identical in all respects except for a small discrepancy in one of the distances will be assigned to different triplet types if the slightly discrepant distances fall either side of a bin boundary. We therefore run the entire overlay generation procedure twice, using different bin definitions. The overlays from the two runs are pooled before filtering. By default, the first set of bins is: 0.5–3.0, 3.0–5.0, 5.0–7.0, 7.0–9.0, 9.0–11.0, 11.0–13.0 Å. The second is: 0.5–3.5, 3.5–6.0, 6.0–8.5, 8.5–11.5, 11.5–13.5 Å. Triplets are ignored if they involve a distance below the lowest bin boundary, or above the highest.

#### Fingerprint calculation

**Fig. 2:**
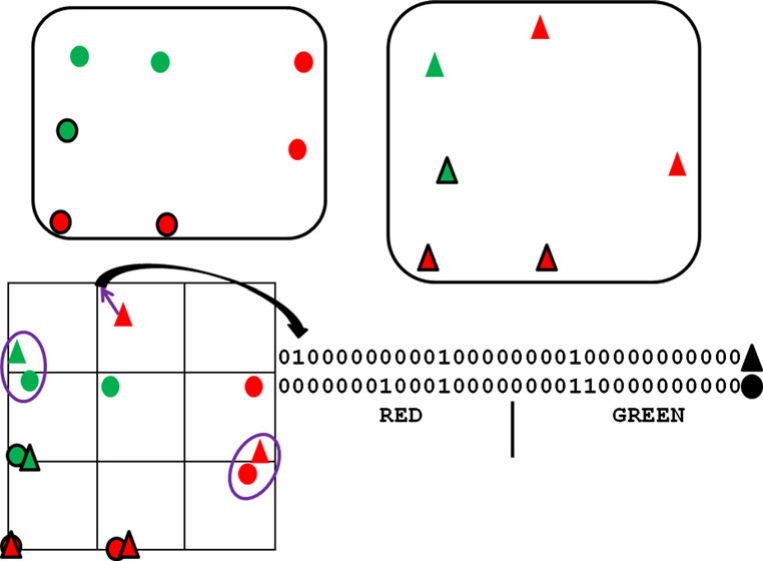
Simplified example of fingerprint algorithm. Two molecules are represented as collections of fitting points, shown as circles for one molecule, triangles for the other. The fitting points represent two types of chemical features, *red* and *green*. The molecules contain approximately congruent triangles of fitting points (outlined in *black*), the “core triplets”. The fitting points of each molecule are oriented so that their core triplets are approximately superimposed, and a grid placed over the resulting fitting-point assembly (*bottom left*). Each fitting point, except those of the core triplets, is mapped to the nearest grid point. For example, the *red triangle* at the *top* is mapped to the grid point shown by the *purple arrow*. A bit string is created for each molecule to represent grid-point occupancy, the first (last) 16 bits capturing occupancy by *red* (*green*) features. For example, the second bit for the *triangle* molecule is set to 1 because the second grid point (counting in *rows* starting at *top left*) is occupied by a *red* fitting point. There are two positions in which both bit strings have on bits, revealing the close proximity of the circled fitting points

For a given iteration step, let the triplet type under consideration be called the base triplet type, and let a triplet belonging to that type be a base triplet. The aim is to perform a multiple alignment of all ligand conformations containing a base triplet so that, for each such conformation, the base triplet is placed in a standard position and orientation. The positions in Cartesian space of all fitting points of the aligned conformations (excluding the base-triplet fitting points) are mapped onto a 3D grid which is converted into a fingerprint (Fig. 2). The fingerprint allows rapid searching for combinations of ligand conformers (one per ligand) whose fitting points occupy similar positions in space.

The algorithm loops over all conformations of all ligands. For each conformation, only the fitting points are considered, not the atoms. If the conformation does not contain a base triplet it is rejected. If it does, the points of that triplet are numbered 1, 2 and 3 by a simple canonicalisation algorithm. (Acceptor points are assigned lower numbers than donor points, and donors lower than hydrophobes. If all three points have the same type, numbering is such that the bins in which the inter-point distances lie are in the order 2–3 ≤ 1–3 ≤ 1–2. When points 1 and 2, but not 3, have the same type, the rule is 2–3 ≤ 1–3; when 2 and 3, but not 1, have the same type, then 1–3 ≤ 1–2.) The rotation/translation transformation is calculated that places the triplet centroid on the origin, point 1 on the +x axis, and point 2 in the xy plane with y ≥ 0. This transformation is applied to all fitting points of the conformation. The resulting fitting-point positions are stored. If the base triplet is degenerate, so that there is no unique canonicalised order, all valid orderings are used in turn, a separate set of fitting-point positions being generated for each. If the conformation contains more than one base triplet, the process is repeated for each in turn.

A 3D grid is constructed, large enough to enclose all the fitting-point positions generated by the above procedure. By default, a grid resolution of 1.5 Å is used. Let the number of points in the grid be G. Each set of fitting points, corresponding to a particular ligand conformation aligned with a base triplet in the standard orientation, is converted to a fingerprint as follows. A bit string of length 3G is created. The first segment of G bits will capture donor fitting-point positions, each bit corresponding to one of the grid points. The other two segments will capture acceptor and hydrophobe-centroid fitting-point positions. All bits are initialised to 0. Each fitting point in the ligand conformation (except those of the base triplet) is mapped to its nearest grid point and to the six adjacent points in the ±x, ±y and ±z directions. Depending on the type of feature that the fitting point represents, the bits corresponding to these seven grid points in the donor, acceptor or hydrophobe segment of the bit string are set to 1. The purpose of setting seven rather than one bit is to smear out the fitting point and hence make the algorithm more forgiving. However, this may be unnecessary as results appear equally good if smearing is switched off (that is, just the bit corresponding to the nearest grid point is switched on).

When all aligned conformations have been processed, the result is a fingerprint table (we call it an alignment fingerprint), each row corresponding to an aligned ligand conformation, each column to a particular grid point and feature type. Empty columns are eliminated. Each row of the table is quite similar to a Bloom fingerprint, as used in the Pharmer program [31], but the rows are not hashed, fitting points can be “smeared” over several bits, and the objective is pharmacophore elucidation rather than the screening of pharmacophore searches.

#### Fingerprint searching

The alignment fingerprint is searched for combinations of rows (one row from each ligand) that have high concordance. This is equivalent to searching for ligand conformations (one per ligand) that, when overlaid by superimposing the base triplet fitting points, have other fitting points close together. Searching for good row combinations is the rate-limiting step of overlay generation. Each trial combination of rows is scored by:

4 $$ {\text{B}} = {\text{wA}} - {\text{O}} $$

A is the number of bits set on in the bit string obtained by logically ANDing the trial set of rows; O is the corresponding quantity for the bit string produced by logical OR; w is an integral weight (default w = 2).

A bit value of 1 in the AND string is suggestive of a full pharmacophore point, since the aligned ligand conformations corresponding to the ANDed rows must all have the same type of fitting point mapped to the same grid point. Thus, large values of A are favourable. Conversely, small values of O are desired, since the more “column sharing” there is (two or more of the selected rows having “on” bits in the same column), the higher the concordance of the selected rows. O is sensitive both to full and partial pharmacophore points. The larger the weight w, the greater the premium placed on full points.

Finding good row combinations is achieved with a greedy algorithm which involves n steps, where n is the number of ligands. In the first step, a starting row is chosen. Selection is biased towards rows containing a large number of “on” bits in highly occupied columns. In the second step, one of the ligands is chosen at random, subject to the constraint that it cannot be the ligand to which the row selected at step 1 belongs. Every row belonging to the second ligand is ANDed and ORed with the starting row, and the one producing the best B value accepted (if there is a tie, one of the tied rows is selected at random). The remaining steps proceed in similar fashion. At each step, rows corresponding to the new ligand are combined with the strings produced by ANDing and ORing all the rows already accepted.

Typically, we generate 200 solutions (row combinations) from each fingerprint. This number is under the user’s control. Each run of the greedy algorithm produces one solution. Thus, if P solutions are required from a fingerprint, they are generated by using P different starting rows, unless P exceeds the number of rows in the fingerprint. In this case, the rows are iterated over again, but using different ligand ordering during the greedy algorithm. The larger P, the more thorough the search. All the solutions from all fingerprints are pooled, giving 10,000 solutions in total when 25 fingerprints are used for each of two distance-bin definitions. The total number will be less than this if fewer than 25 fingerprints can be constructed for either set of distance bins; this will occur if fewer than 25 triplet types occur in all of the ligands. The solutions are stored as chromosomes. In each chromosome, the mapping table contains the indices of the fitting points comprising the base triplets of the rows in the solution, and the conformer indices reflect the ligand conformations from which the rows were constructed. Each chromosome can be used to construct the corresponding molecular overlay.

#### Stepwise approach

A limitation of the method is that overlays can only be generated from base triplets that occur in at least one conformation of every ligand. If there is no such base triplet, one possible remedy is to create overlays in stepwise fashion. The overlay generation is first run on a subset of ligands which do share a common base triplet. After filtering, this will result in several overlays of the subset of ligands. The program can treat these as “conformations” of a “supermolecule”, for each of which fitting points are placed to represent the features of all the ligands in the overlay. Where fitting points of the same type from different ligands are close together, they are merged into a single, average point. This is done by leader cluster analysis (see above). By default, points separated by >1.5 Å will not be placed in the same cluster and therefore will not be merged. Because the supermolecule has more fitting points than any of the individual ligands from which it is comprised, there is an increased chance of finding common base triplets between it and the remaining ligands. Overlays of the complete set may therefore be built up by a succession of steps. Users must specify the number and nature of the steps in a stepwise overlay generation. A step can involve overlaying supermolecules on other supermolecules. For example, when overlaying ligands L1, L2, L3 and L4, a typical step specification might be: L1 on L2 to give supermolecules (L1 + L2); L3 on L4 to give supermolecules (L3 + L4); (L1 + L2) on (L3 + L4) to give the final overlays. A step can involve both supermolecules and ordinary molecules; for example: L1 on L2 to give (L1 + L2); (L1 + L2) on L3 and L4 to give the final overlays.

#### Constraints

The algorithm can be used to generate constrained overlays. For example, suppose all the ligands contain a quaternary nitrogen atom and the user is only interested in overlays in which these atoms are superimposed. An artificial “constraint” feature type is introduced, to which only the quaternary nitrogen atoms belong, a fitting point being placed on each. During triplet enumeration, triplets which do not contain one of these fitting points are rejected. The ensuing fingerprint algorithm is therefore constrained to produce only solutions which superimpose the quaternary nitrogen atoms.

### Overlay filtering

Filtering aims to select a diverse subset of the best of the generated overlays. The procedure begins by scoring the overlays, using some or all of the objective functions described earlier, either separately or as a weighted linear combination. By default, we use volume, hydrogen bond and hydrophobic scores, but not energy, as all ligand conformations in the generated overlays will have been taken directly from the conformers supplied by the user. The objective functions are computed separately and converted to a single number by Fonseca-Fleming Pareto ranking [32]. Overlays whose Pareto rank exceeds a threshold (set by default to 5) are rejected.

When Pareto ranking, we usually set score constraints. In unconstrained Pareto ranking, one solution will be deemed to dominate another if, and only if, it scores better on at least one objective and does not score worse on any objective. When a score constraint is applied (for example, V < 900), an extra rule is invoked: for any pair of solutions, if one breaks a constraint (for example, V = 901) and the other does not, the solution breaking the constraint is deemed to be dominated by the other. Score constraints can be specified in absolute or percentile terms. By default, we use the latter, requiring that an overlay must be in the best 30 % of volume scores and the best 30 % of hydrogen-bond scores to avoid breaking a constraint.

Typically, we limit the final number of solutions after filtering to ≤20. Thus, if application of the Pareto rank threshold leaves too many solutions, they are further reduced in number as follows. They are ordered on their Borda tallies (the sum of the ranks of the individual objective scores [33]). The highest ranking solution (best on Borda tally) is chosen to be part of the final solution set. Solutions similar to this one are rejected. The best solution of those that remain is chosen, similar solutions rejected, and so on until the required number of solutions has been chosen or the solutions are exhausted. Similarity is measured by the consensus coefficient described later; solutions are rejected if their dissimilarity from any overlay already accepted is <0.05.

### Overlay refinement

This has the purpose of improving an already good overlay from the preceding steps by bringing approximately overlaid groups into tighter alignment. Refinement can be achieved by simulated annealing, randomly changing one ligand torsion value, or applying a small random rigid-body translation or rotation to one ligand, in each step. Changed torsion values must respect any torsion-angle constraints set by the user. The following cost function usually gives acceptable results:

5 $$ {\text{F}} = {\text{HB}} - 0.5{\text{V}} + {\text{HY}} - 0.3{\text{E}} $$

The initial annealing temperature is typically set to a low value, because the aim is to achieve minor improvements rather than perform a wide exploration of overlay space.

Annealing is usually successful at producing well-refined solutions, but it is slow. We have recently developed a much faster method using gradient-based optimisation, full details of which will be published in a subsequent paper.

### Overlay multiplication

**Fig. 3:**
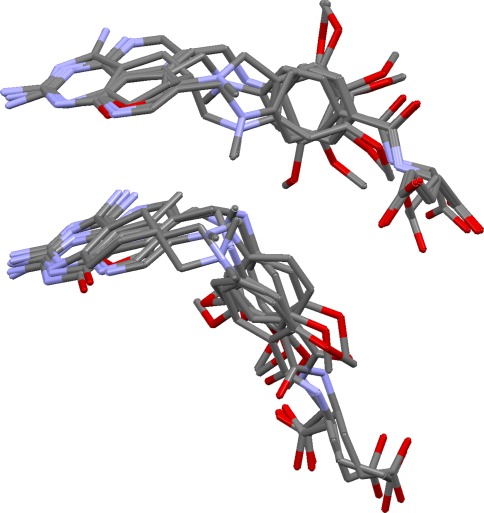
Two overlays of dihydrofolate reductase ligands with identical feature mappings but different ligand conformations (and therefore leading to different pharmacophore queries)

This aims to take a good overlay and investigate whether other alignments exist with the same mapping of features but different ligand conformations (Fig. 3). This is often possible if the ligands are flexible, and of practical concern if the aim is to produce a pharmacophore query for virtual screening. Ideally, all possible solutions should be found at the overlay generation stage, but only a limited number of solutions will normally be requested, and the diversity algorithm used during filtering may bias selection towards overlays expressing different feature mappings.

The multiplication procedure is related to the algorithm described in our earlier publications [17–19]. The first step is to construct a chromosome mapping table that reflects all the full and partial pharmacophore points in the starting overlay. A population of 150 chromosomes is set up, each containing this mapping table but with randomised torsion-angle values. The population is subjected to MOGA optimisation. 150 children are produced in each generation by torsion mutations, torsion crossovers, or small mutations to the rigid-body ligand rotation or translation data. Each mutation is restricted to a single torsion value or to the rigid-body translation or rotation data of a single ligand. In each torsion crossover, the swap is restricted to torsion angles involving a single ligand. Mutated torsion values must respect any torsion-angle constraints set by the user. Parents are chosen by tournament selection.

At each generation, parent and child populations are merged and Pareto ranked, using the objective functions V, HB, HY and E. Up to 150 chromosomes from the merged population are accepted for the next generation. Selection is based on Pareto ranks, with niching to promote geometric diversity. Chromosomes are placed in the same niche if the dissimilarity of the overlays for which they code is less than a set value. Once a niche is full, no further chromosomes that would belong to that niche can be accepted. For speed, dissimilarity is measured not by the coefficients described below but by the following crude technique. A subset of atoms is chosen, including one from (or very near to) every feature of every ligand. For each overlay, the matrix of squared distances between the chosen atoms is calculated. The dissimilarity of an overlay pair is determined by the mean absolute difference between corresponding elements of their squared distance matrices.

### Solution analysis

The following methods were programmed to aid comparison of the overlays produced for a set of ligands.

#### Overlay dissimilarity coefficients: introduction

Two questions are relevant when comparing a pair of overlays of the same ligands. First, are the same ligand groups matched (that is, how different are the pharmacophores in terms of the number and types of pharmacophore points they contain, and the individual ligand fitting points that contribute to them)? Second, are the overlays similar geometrically? We therefore use three dissimilarity measures, one pharmacophore based, one based on geometry, and the third a consensus measure.

#### Pharmacophore dissimilarity coefficient

The pharmacophore present in each of the overlays (A, B) to be compared is identified by cluster analysis of the ligand fitting points. All pairs of pharmacophore points, one from A and one from B, that are of the same type (all donor pairs, all acceptor pairs, and so on) are examined. For a given pair, P_A_ and P_B_, let N_A_ be the number of ligand fitting points in P_A_, N_B_ be the number in P_B_, and N_AB_ be the number that are in both P_A_ and P_B_. The similarity of the pair is computed by the Tanimoto metric T = N_AB_/(N_A_ + N_B_ − N_AB_). The weight of the pair is defined as w = [(N_A_ + N_B_)/2]^2^.

Pharmacophore points in A are then matched with those in B, by first matching the pair with the highest Tanimoto coefficient, then the pair with the next highest (excluding any pair involving a pharmacophore point that has already been matched), and so on. Some pharmacophore points may be left unmatched. For these, the quantity U = ΣN_i_^2^ is calculated, where summation is over the unmatched pharmacophore points and N_i_ is the number of ligand fitting points in the ith unmatched point. The pharmacophore dissimilarity coefficient, D_p_, is calculated as

6 $$ {\text{D}}_{\text{p}} = 1 - (\Upsigma {\text{w}}_{\text{i}} {\text{T}}_{\text{i}} )/({\text{U}} + \Upsigma {\text{w}}_{\text{i}} ) $$

the summations being over the matched pairs.

#### Geometric dissimilarity coefficient

The geometric dissimilarity of overlays A and B is quantified by least-squares fitting a selection of atoms in A, chosen to include one atom from (or very near to) every feature of every ligand, onto the corresponding atoms in B. To allow for local topological symmetry, a two-step procedure is used. In step 1, the selected atoms of each ligand in A are least-squares fitted onto the selected atoms of the corresponding ligand in B, using all possible ways of matching the atoms (given that there may be many ways of matching the ligand graph onto itself). For each ligand, the atom pairing giving the lowest root mean square deviation (rmsd) is stored. In step 2, A is least-squared fitted onto B, using the atom pairings stored from step 1. The interatomic distance of each matched atom pair in the superposition of A and B is converted to a normalised quantity q by the transformation:

$$ {\text{q}} = 0\quad {\text{if}}\; {\text{d}} < 0.5\,{\text{\AA}};{\text{ q}} = ({\text{d}} - 0.5)/(3.5 - 0.5)\quad {\text{if}}\; 0.5 \le {\text{d}} \le 3.5\,{\text{\AA}};{\text{ q}} = 1\quad {\text{if}}\; {\text{d}} > 3.5\,{\text{\AA}} $$

The dissimilarity coefficient, D_G_, is the average of the q values.

#### Consensus dissimilarity coefficient

The consensus dissimilarity, D_C_, is √(D_P_D_G_).

#### Superposition of overlays

Any two overlays can be superimposed automatically to aid their comparison. Superposition is achieved either by least-squares fitting of atoms or of pharmacophores (using pharmacophore-point pairings derived from calculating the D_P_ coefficient). If the latter is used, the consensus pharmacophore of the two solutions is also calculated and displayed.

#### Mapping of overlays

**Fig. 4:**
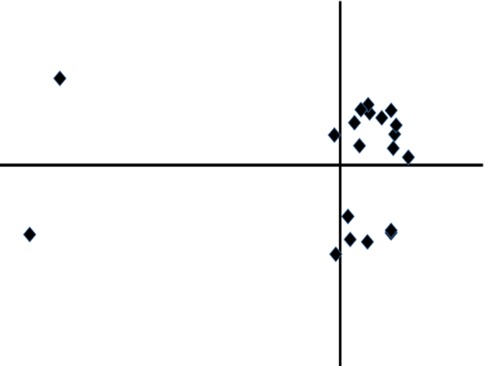
Plot of overlays of cycle checkpoint kinase ligands, revealing that the overlays fall into two distinct clusters, with two outliers (the *horizontal* and *vertical* axes represent the first and second dimensions, respectively, from the multidimensional scaling calculation)

Multidimensional scaling (performed with the SMACOF algorithm [34]) is used to produces 2D or 3D plots of the final set of overlays, the intention being that similar overlays should lie close together on the plot [35]. Three separate plots are calculated, based on each of the dissimilarity coefficients described above. Plots can be coloured on any of the objective scores. The plots can be very revealing. For example, Fig. 4 shows that solutions fall into two main clusters, with two gross outliers. However, overlays are complex objects and variations between them can be represented only approximately in low dimensional space. Thus, while useful, the plots should not be over-interpreted.

### Selection of parameter values

Parameters used in the hydrogen bond and hydrophobic scores were chosen so that the relative contributions to these scores of the various clusters of donors, acceptors and hydrophobes in the true overlays of the test set seemed reasonable, in our subjective judgement. The extent to which vdw radii were reduced for calculation of the energy score, and the strategy of ignoring the worst contact provided it was less repulsive than 150 kcal/mol, were chosen to give good discrimination between the calculated energies of (a) OMEGA-generated and (b) randomly-generated conformations of the test-set ligands. The premium placed on full pharmacophore points when searching alignment fingerprints (w in Eq. 4), the number of fingerprints used, the number of overlays generated per fingerprint, and the filter thresholds and score constraints, were selected by manual experimentation on two of the ten sets of ligands used in the validation (neprilysin and dihydrofolate reductase ligands).

## Validation

### Test data

The program was tested on ten sets of protein–ligand complexes from the PDB (Table 1), and on some subsets thereof (Table 2). All complexes are members of the Astex Non-Native Set, which was compiled from well-refined structures with a bias towards therapeutically relevant proteins [36]. The complexes in each set were superimposed by least-squares fitting the binding-site atoms in Relibase+ [37], hence producing the true, crystallographically-observed overlay. Each complex was inspected to establish probable ligand protonation states and identify protein–ligand interactions. This enabled the true pharmacophore points to be determined (clusters of atoms or groups that form common interactions with the protein) and distinguished from incidental clusters of donors, acceptors and hydrophobes (for example, clusters of acceptors or donors that interact only with solvent).

**Table 1:** Test sets

Protein	Number of complexes	PDB codes
Protein kinase 5 (PK5)	2	1v0o, 1v0p
Fatty acid binding protein (FABP)	3	1tou, 1tow, 2hnx
Neprilysin (NEP)	4	1dmt, 1r1h, 1r1j, 1y8j
Dihydrofolate reductase (DHFR)	6	1drf, 1hfr, 1mvt, 1pd9, 1s3v, 2dhf
Checkpoint kinase (Chk1)	16	1nvq, 1nvr, 1nvs, 1zlt, 1zys, 2br1, 2brb, 2brg, 2brh, 2brm, 2bro, 2c3l, 2cgu, 2cgw, 2cgx, 2hog
Neuraminidase (NEU)	11	1a4g, 1a4q, 1b9s, 1b9t, 1b9v, 1inf, 1inv, 1ivb, 1nsc, 1nsd, 1vcj
Carbonic anhydrase (CA)	13	1bn3, 1bn4, 1bnq, 1cim, 1eou, 1if7, 1oq5, 1xpz, 1zgf, 1zh9, 2eu3, 2hoc, 2nng
Adenosine deaminase (ADA)	11	1krm, 1ndv, 1ndw, 1ndy, 1o5r, 1qxl, 1uml, 1v7a, 1v79, 1wxy, 2e1w
Heat shock protein 90 (HSP)	10	1byq, 1uy8, 1yc1, 1yc4, 1yet, 2bsm, 2byi, 2bz5, 2cct, 2uwd
Acetylcholinesterase (AChE)	11	1dx6, 1e66, 1eve, 1gpk, 1gpn, 1h23, 1w4l, 1zgb, 2ack, 2c5g, 2ckm

**Table 2:** Test subsets

Protein/subset	Number of complexes	PDB codes
ADA/1	10	1ndv, 1ndw, 1ndy, 1o5r, 1qxl, 1uml, 1v7a, 1v79, 1wxy, 2e1w
ADA/2	4	1o5r, 1qxl, 1uml, 1wxy
ADA/3	4	1ndv, 1o5r, 1qxl, 1uml
HSP/1	7	1yc1, 1yc4, 2bsm, 2byi, 2bz5, 2cct, 2uwd
HSP/2	3	1byq, 1uy8, 2cct
AChE/1	9	1dx6, 1e66, 1gpk, 1gpn, 1h23, 1w4l, 1zgb, 2ack, 2ckm
AChE/2	4	1h23, 1w4l, 1zgb, 2ckm

Ligand models were created with CORINA [38] with addition of required hydrogen atoms. Six sets of conformers were generated for all ligands. Three (RAW5000, RAW1000 and RAW200) were produced using the raw CORINA-generated molecules as input, with the maximum number of conformers per ligand set to 5,000, 1,000 and 200, respectively. The OMEGA rms and ewindow parameters were set to 0.5 Å and 10 kcal/mol, respectively, and the –fromCT flag set to false; default values were used for other parameters. The remaining conformer sets (OPT5000, OPT1000 and OPT200) were generated in similar fashion except that the CORINA models were subjected to geometry optimisation with the SZYBKI molecular mechanics program [39] before input to OMEGA. OMEGA changed the bond types of a small number of chemical groupings (in particular, removing the formal charge on some aromatic nitrogens by making the ring non-aromatic) but we felt the error was sufficiently unimportant that it could be ignored (a conservative decision, since sub-optimum bond types are likely to worsen rather than improve validation results). A table is included in the Supporting Information giving, for each ligand, the non-hydrogen atom rmsd between the binding conformation and the closest approximation to that conformation in each conformer set. In general, these are satisfactorily small.

### Success criteria

Choosing success criteria is not easy. First, it cannot be assumed that the true overlay is the most plausible way in which the ligands can be aligned. It is possible that an incorrect solution may look more convincing and such a solution should be presented to the user for consideration. Second, it is more important to correctly predict the feature mappings than the ligand conformations. An overlay with correct feature mappings is very useful, even if the ligand geometries are wrong, because: (a) it indicates which functional groups are important for binding; (b) the pharmacophore query it suggests will probably find useful hits (since flexible active molecules in the search database may be able to adopt the conformations required to match the query); (c) it can serve as a starting point for overlay multiplication. Third, the all-atom rmsd from the true overlay is a poor success measure, since it can be low even if the prediction has serious faults [15]. We therefore define success as generating an overlay with substantially correct feature mappings (preferably, but not necessarily, in their correct relative positions) in the small number of highly ranked solutions that a user is likely to view (we have assumed users will inspect up to 20).

**Fig. 5:**
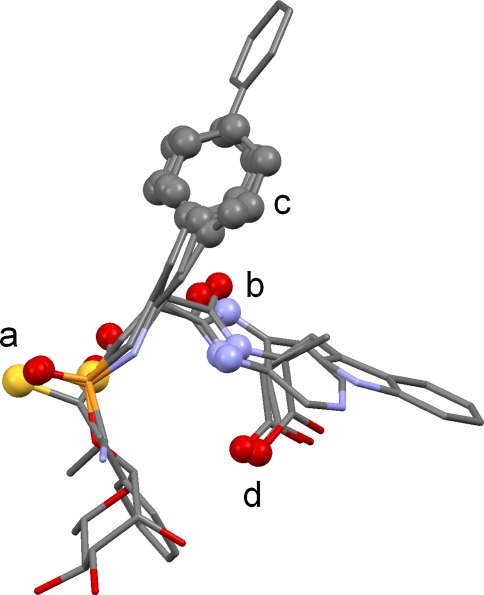
True overlay of neprilysin ligands showing the pharmacophore-point groups (in *ball-and-stick style*) referred to in the text

The pharmacophore points (full and partial) in each true overlay were manually divided into groups. For example, those of the NEP ligands (Fig. 5) were divided into: (a) the acceptor points representing the acidic atoms that coordinate the active-site zinc and hydrogen bond to His711 and Glu584; (b) the pair of donor and acceptor points representing amide and imidazo groups that hydrogen bond to Asn542 and Arg717; (c) the hydrophobic point representing phenyl and isobutyl groups that interact with Val580, Trp693 and other nearby residues; (d) the acceptor point corresponding to carboxylate oxygen atoms that hydrogen bond to Asn542. Each pharmacophore-point group was classified as being of major, moderate or minor importance, depending on whether it contains full pharmacophore points, partial points involving several of the ligands, or partial points involving only a small number of ligands. Table 3 summarises the pharmacophore-point groups for the NEP ligands. Analogous tables for other test sets are available as Supporting Information, together with annotated ligand chemical diagrams.

**Table 3:** Pharmacophore-point groups for the neprilysin ligands

Group	Description	Full^a^	Partial^b^	Importance
a	Acidic groups (including thiolates) binding Zn, accepting from E584, H711	1	1	Major
b	Amide and imidazo groups donating to N542, accepting from R717	2	0	Major
c	Phenyl and isobutyl groups making hydrophobic contacts in vicinity of V580, H583 and W693	1	0	Major
d	Carboxylate oxygens accepting from N542	0	1	Moderate

^a^Number of full pharmacophore points in group

^b^Number of partial pharmacophore points in group

We then identified the atoms (donors, acceptors and dummy atoms at the centroids of hydrophobes) that constituted the pharmacophore points in each group (for example, the carboxylate oxygens constituting group d of the NEP ligands). For any given predicted overlay, we calculated the quantities R_i_, i = 1, 2,…, N, where N is the number of pharmacophore-point groups and R_i_ is the rmsd obtained when the atoms constituting the pharmacophore points of the ith group in the true overlay are least-squares fitted onto the corresponding atoms in the predicted overlay. We also calculated R_total_, the rmsd obtained when all atoms of the true overlay that were included in any R_i_ calculation are least-squares fitted onto the corresponding atoms of the prediction. If all R_i_ are small, the predicted overlay has all the correct pharmacophore points but not necessarily in their correct relative positions (that is, correct feature mappings but possibly incorrect ligand conformations). If both R_total_ and all the R_i_ are small, the predicted overlay has correct feature mappings and ligand conformations. In addition to these objective measures, we also assessed solutions subjectively by superimposing them on the true overlay and manually looking for misplaced ligands.

## Results

Up to 20 solutions were produced for each test set, including the subsets in Table 2. RAW5000 conformers were used as input and all program parameters were set at their default values. No overlay refinement or multiplication was performed unless otherwise stated below. The solutions in each set were ranked from 1 to 20, based on their Borda tallies for the V, HB and HY objective functions (see Overlay Filtering). Each solution was characterised by its R_i_ and R_total_ values and many were manually inspected. Table 4 summarises the results. The R_i_ and R_total_ values (in Å) pertain to the solution that appeared to us to best represent the true overlay. Successive R_i_ values on each line correspond to pharmacophore-point groups a, b, c, etc. in Table 3 and the analogous tables in Supporting Information, and are separated into those that represent groups of major, moderate and minor importance. The number of seriously misplaced ligands (if any) in the solution is given, together with the solution rank (column headed “rank, best”). The table also shows the rank of the highest-ranked solution that had substantially correct feature mappings but not necessarily the correct geometry (“rank, highest”). For some sets (listed at the foot of the table), none of the solutions were considered satisfactory; R_i_ and R_total_ are not given in these cases.

**Table 4:** Results obtained from RAW5000 conformers

Set or subset	R_i_ (major)	R_i_ (moderate)	R_i_ (minor)	R_total_	Seriously misplaced	Rank, best	Rank, highest
PK5	0.6	–	–	0.6	0	2	1
FABP	0.3, 0.6, 1.5, 1.7	–	–	1.5	0	5	1
NEP	1.6, 0.4, 0.5	0.4	–	1.2	0	2	1
DHFR	0.4	1.3	0.2, 0.2	2.0	0	2	2
Chk1	0.5	1.2, 1.0	0.3, 2.1	1.2	0	1	1
NEU	0.7, 0.3	0.4	0.9, 0.6, 0.9	0.8	1	4	1
CA	1.3, 0.5	1.6, 1.6	1.4	1.8	2	4	4
ADA/1^a^	–	0.6, 0.5, 0.3, 0.7, 1.1	1.9	1.3	0	1	1
ADA/2	0.9, 0.7, 0.2, 1.3	0.2, 0.2	–	1.9	0	18	1
ADA/3	0.9, 0.8, 0.7, 1.7	0.1, 0.2	–	2.5	0	3	3
HSP/1	0.5, 0.7, 0.2	0.2, 0.2, 0.9	0.2	1.0	0	5	1

No satisfactory solutions were obtained for ADA, HSP, HSP/2; AChE, AChE/1, AChE/2

^a^Obtained by stepwise method

## Discussion

Discussion is confined to the results from the RAW5000 conformer set, with a brief summary of the effects of using other conformer sets at the end.

### Protein kinase 5

**Fig. 6:**
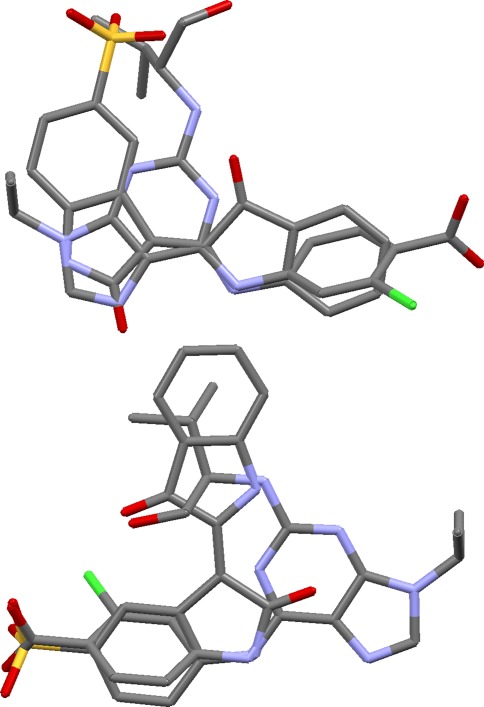
Two predictions for protein kinase 5 ligands. The *top* one is similar to the true overlay, the *bottom* one is a credible alternative with ligand acid groups overlaid

The main problem with this otherwise simple test set is that both ligands contain acid groups, and we might expect the algorithm to produce overlays in which these groups are superimposed. However, the acid groups are widely separated in the true overlay, neither forming any significant interactions with the protein. Ideally, the program should generate both the correct solution and the obvious but incorrect alternative. This is achieved. Only nine solutions survive the filtering, of which six are closely related and similar to the true overlay, while the other three have the acid groups superimposed (Fig. 6). The occurrence of clusters of similar solutions in the filtered output is a consequence of using a low filtering dissimilarity threshold (0.05), which tends to focus in on the most convincing overlays at the expense of diversity. The optimum value of this parameter may well vary from case to case, depending, for example, on how many pharmacophore queries the user wishes to generate.

### Fatty acid binding protein

**Fig. 7:**
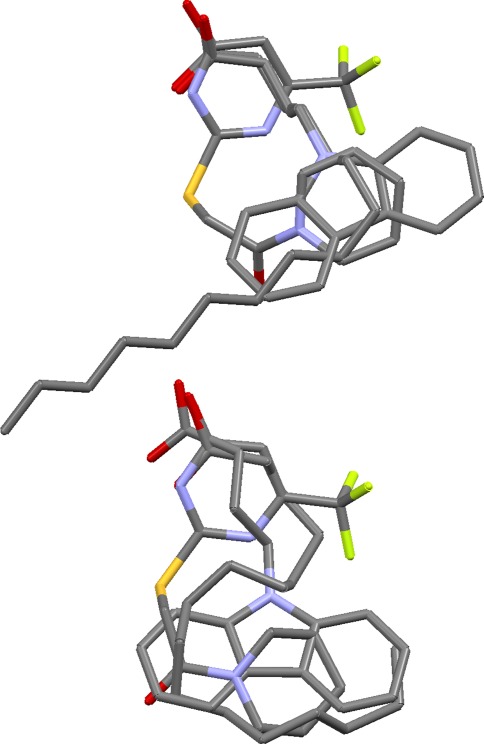
Top ranked solution for fatty acid binding protein ligands before (*top*) and after (*bottom*) refinement

This is a simple set, the only challenge being to find the folded conformation of the C15 chain of the 2hnx ligand that places it within the envelope of the other two ligands. This is not very well achieved in the overlay generation step, which tends to produce solutions like that shown at the top of Fig. 7 (solutions from OPT5000 conformers tend to be better). However, refinement by annealing readily fixes the problem (Fig. 7, bottom). When applied to the top-ranked solution, refinement reduces an initial overlay volume of 451 to 363 Å^3^, marginally lower than that of the true overlay. A minor but understandable error in the hydrogen-bond matching is that the carboxylate oxygens of the 2hnx and 1tow ligands tend to be matched onto both the hydroxyl oxygen and one of the pyrimidine nitrogens of the 1tou ligand. In fact, only the hydroxyl oxygen of the latter ligand appears to hydrogen bond to the protein. It is not uncommon for the algorithm to find better matching of hydrogen-bond groups than occurs in reality.

**Fig. 8:**
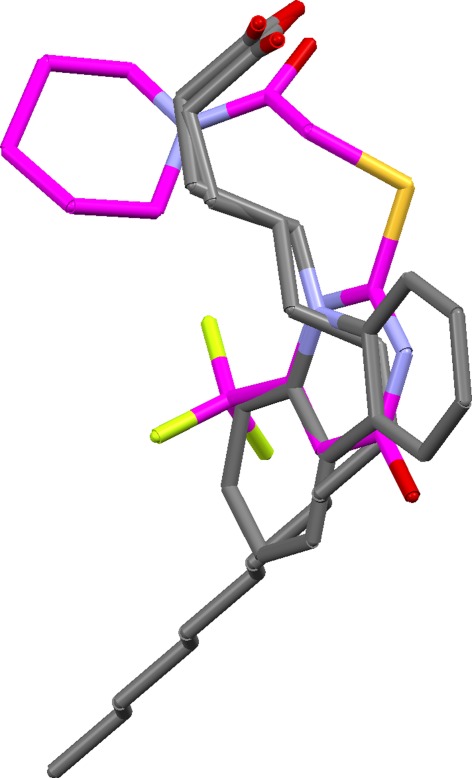
Constrained overlay of fatty acid binding protein ligands (carbon atoms of 1tou ligand shown in *magenta*)

Figure 8 shows the top-ranked solution when the carbonyl oxygen of the 1tou ligand is constrained to superimpose on carboxylate oxygen atoms of the other ligands. This produces a fundamentally different, but still credible, overlay. Constrained overlaying is an easy way to explore specific overlay hypotheses.

### Neprilysin

**Fig. 9:**
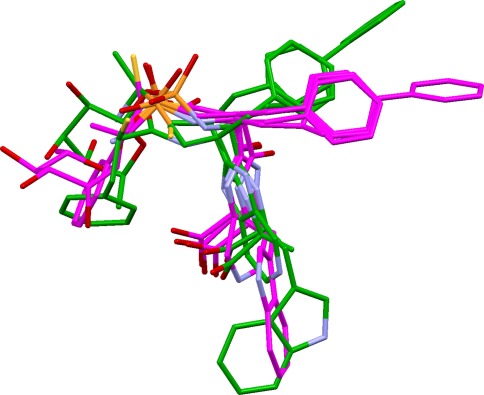
Second-ranked solution for neprilysin ligands (carbons coloured *green*) superimposed on true overlay (carbons in *magenta)*

This set contains only four ligands, but they are flexible and feature rich and therefore moderately challenging to overlay. The solutions are excellent. Eight of the top ten solutions have essentially correct feature mappings, though several involve different ligand conformations from those seen in the crystal structure. There are invariably minor errors in the vicinity of the zinc-binding groups but never sufficient to obscure their obvious significance to binding. The second-ranked solution has both correct mappings and almost correct conformations (Fig. 9). Solution 9 is a different but credible overlay in which the thiolate of the 1y8j ligand is matched with carboxylate oxygens from the other ligands. Only a small minority of the top twenty solutions look unconvincing.

The 1r1j and 1y8j ligands in this set were built in their thiolate forms, since it was known that the sulfur atoms coordinate zinc and are likely to be ionised. Solutions generated from the unionised forms would probably have been much poorer since the sulfur atoms would not have been regarded as acceptors. In a genuine drug discovery project, the quality of answers would therefore depend on whether investigators were aware of the probable presence of a zinc ion in the binding site of the target protein.

### Dihydrofolate reductase

Results on this set are good. The critical requirement is to generate the correct alignment of heterocycles, in which some of the bicyclic systems are flipped in order to achieve the required matching of hydrogen bonding groups. The correct heterocycle alignment is present in about half of the solutions that survived filtering. Most of the remainder contain plausible alternative alignments. The highest ranked solution with the correct heterocycle alignment (solution 2) also has the amide and carboxylate groups of the 1drf, 1hfr and 2dhf ligands correctly superimposed and all six phenyl-ring centroids in roughly the same position. In the true overlay, there is a distinct separation between the phenyl groups of the 1drf, 1hfr and 2dhf ligands and those of the remaining ligands.

Although solution 2 has almost perfect feature matching, it has incorrect ligand conformations (extended rather than bent). This is typical for these ligands: prediction of the correct feature matching is easily achieved but almost always with extended conformations. Only with OPT5000 conformers can solutions be generated with ligand conformations similar to those seen in the crystal structures. (This is probably due to the fact that the RAW5000 conformers do not contain good approximations of the binding conformations of the 1drf and 2dhf ligands whereas the OPT5000 conformer sets do.) However, the problem is solved by overlay multiplication. Thus, when solution 2 was subjected to this process, both bent and extended overlays with correct feature matching were produced. Figure 3 shows the original solution 2 (top) and the top-ranked solution from overlay multiplication, after refinement (bottom). The latter is close to the true overlay in both feature matching and ligand conformations.

### Checkpoint kinase

**Fig. 10:**
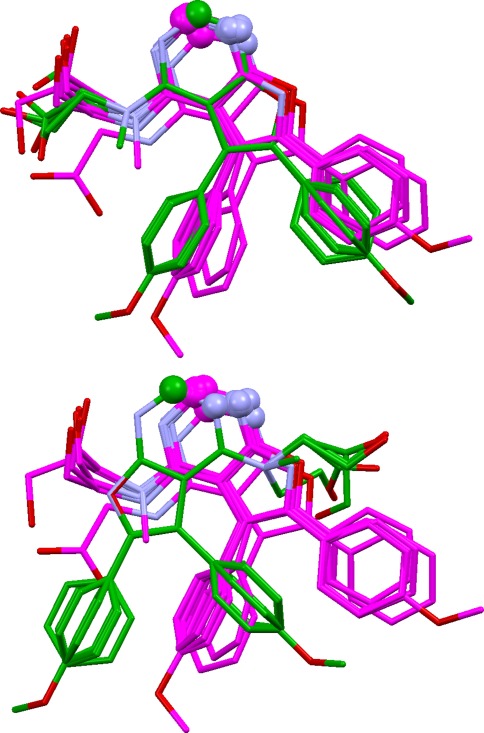
*Top*: the true overlay of the ligands from the checkpoint kinase complexes 2br1, 2brb, 2brg, 2brh, 2brm, 2bro (carbons in magenta) superimposed on the corresponding ligands from one of the correct solutions obtained for the Chk1 test set (carbons in *green*, remaining ligands in set omitted for clarity). *Bottom*: the same true overlay superimposed on ligands 2br1… 2bro from one of the incorrect outlier solutions. Both have the requisite clusters of donor and acceptor atoms (shown as *spheres*) but the acceptor cluster in the incorrect solution involves the wrong pyrimidine nitrogen

This set includes six very similar ligands (2br1, 2brb, 2brg, 2brh, 2brm, 2bro) but is otherwise quite diverse. Results are very good. Most solutions are close to the true overlay and, in particular, the key pair of donor and acceptor pharmacophore points is found, with all the correct donors and acceptors from the individual ligands. It is to this set that the plot shown in Fig. 4 pertains. The two large clusters on the right of this plot comprise essentially correct solutions, the difference between them being that one of the ligands (1zys) is rotated by about 30° in one cluster compared to the other. The two outliers on the left of the plot correspond to a different and incorrect positioning of the six similar ligands referred to above. In both correct and outlier solutions, the CH groups at the 2-position of the fused pyrimidine rings of these ligands are correctly placed in the key donor cluster but, in the outliers, the six ligands are flipped so that the wrong pyrimidine nitrogen is placed in the key acceptor cluster (Fig. 10). In addition, one of the outliers also has another ligand (1zlt) misplaced, resulting in a reasonable but incorrect matching. While the multidimensional-scaling plots are not always useful, they can, as in this case, highlight differences between overlays that might otherwise be missed.

### Neuraminidase

**Fig. 11:**
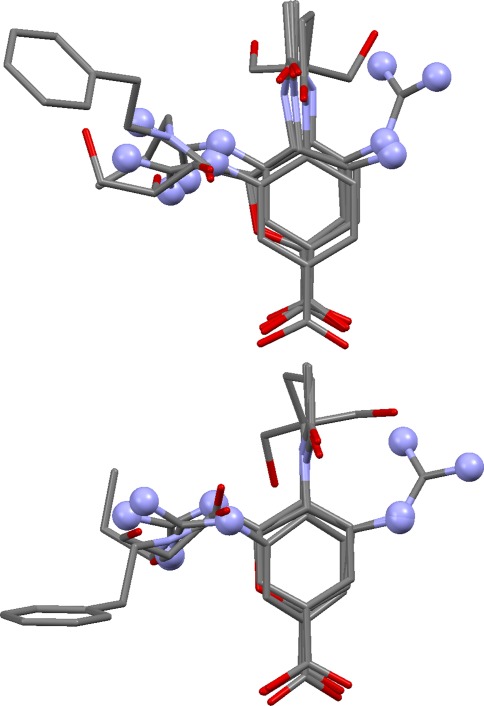
At *top*, the true overlay of four of the neuraminidase ligands (1a4g, 1a4q, 1b9t, 1inf); at bottom, the positions of the same ligands in the best predicted overlay (remaining ligands in set omitted for clarity). In both, two cationic groups (shown in ball-and-stick style) lie on one side of the overlay and two on the other

The ligands in this set are all very similar, each containing a core substructure comprising a 6-membered ring with para-related acid and amide (or lactam) substituents. It was included in the validation to mimic the type of set that might be overlaid in a 3D QSAR exercise. Although the problem may appear trivial, it is not. Accepting that the core substructures from the eleven ligands should be superimposed, there are still many ways of achieving this. Assume the first ligand is placed arbitrarily. Since the bond between the 6-membered ring and the amide or lactam group is rotatable, the second and subsequent ligands may each be superimposed on the first in two ways whilst keeping the core substructures well aligned, giving a total of 2^10^ possibilities for the complete overlay. With this in mind, we were surprised to find that several of the solutions, including the top-ranked, have each molecule “the right way round”. This is particularly impressive as it places two cationic (guanidinium) groups on one side of the overlay and two (a guanidinium and ammonium) on the other, a correct but perhaps surprising arrangement (Fig. 11). The correct solution has a particularly low union volume, which probably explains why it can be found so readily.

The ligand from 1nsc cannot be placed optimally because OMEGA does not generate the rather strained ring conformation reported in the PDB structure. Instead, it generates chair conformers with the carboxylate group axial, meaning that this group is not superimposed on the acid groups of the other ligands. This problem is found with all the conformer sets.

### Carbonic anhydrase

**Fig. 12:**
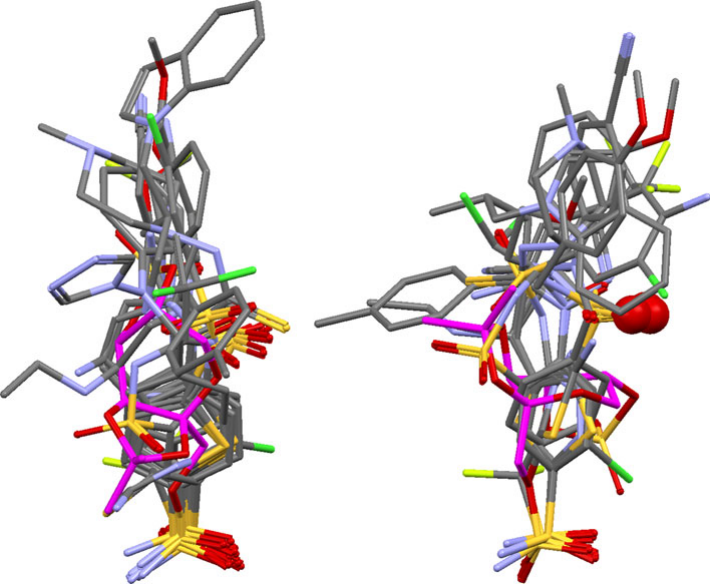
True (*left*) and best predicted overlay (*right*) of carbonic anhydrase ligands. In the prediction, metal coordinating groups are overlaid correctly, and some of the groups hydrogen bonding to Gln92 are properly superimposed (shown as *spheres*), but hydrophobic portions of the ligands are not correctly positioned and the 1eou ligand (carbons in *purple*) is badly misplaced. Overall, it is a rather poor result

These ligands all contain sulfonamide or sulfamate groups that coordinate the active-site zinc atom. Although the R_i_ and R_total_ values for the best solution look reasonable (Table 4), it is not a particularly good prediction (Fig. 12). The metal-coordinating warheads are correctly overlaid (this is true in all solutions) and there is enough of a cluster of acceptors to indicate the presence of the partial pharmacophore point corresponding to interaction with Gln92. The hydrophobic side chains of the 1bn3, 1bn4, 1bnq, 1if7, 1oq5, 1xpz, 1zh9 and 2hoc ligands are overlaid about as well as in the true overlay (which is to say, not very), but in the wrong position (that is, the ligand conformations are different from those in the true overlay). The 1eou ligand is badly misplaced (as it is in all solutions). Overall, the predictions for this set, while containing some of the characteristics of the true overlay, add nothing to what is likely to be discerned by a competent modeler, in contrast to the neuraminidase set, where we feel the program genuinely adds value. Refining the solutions arguably improves the situation somewhat. We have no insight into why the algorithm finds this set comparatively difficult, save to note that the peripheral hydrophobic groups in the true overlay (those remote from the warhead groups) are not tightly overlaid.

### Adenosine deaminase

**Fig. 13:**
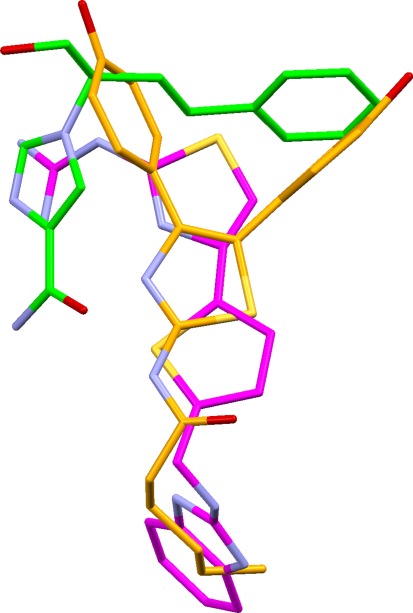
True overlay of three adenosine deaminase ligands from PDB complexes 1ndw, 1ndv and 1wxy (carbon atoms coloured in *green*, *magenta* and *orange*, respectively)

The correct solution is not found for the complete set. An obvious reason is that the ligand from the 1krm complex adopts a binding mode that is drastically different from those of the other ligands. When this ligand is omitted (subset ADA/1), correct solutions are still not generated by the standard approach. All but two of the molecules in the subset are chemically similar imidazoliums, and these are overlaid with ease, but the correct positioning of the other two ligands (1ndv and 1wxy) remains elusive. The problem is that, in the true overlay of the 1ndv and 1wxy ligands on any of the imidazoliums (the 1ndw ligand, for example; Fig. 13), there is no protein residue that hydrogen bonds to all three ligands and the overlap of hydrophobic groups is poor. Thus, no base triplet exists from which an alignment fingerprint can be built to generate the correct answer. The algorithm does find solutions acceptably close to the true overlay for subsets comprising three of the larger imidazoliums and one or other (but not both) of the 1ndv and 1wxy ligands, for which subsets (ADA/2 and ADA/3) a common pharmacophore of size 3 exists in the true overlays.

The correct overlay for ADA/1 can be generated by the stepwise approach. In our first experiment, the imidazoliums were overlaid without the 1ndv and 1wxy ligands. The top 20 solutions were treated as “conformers” of a “supermolecule” and used as input to a second overlay-generation job in which the 1wxy ligand was introduced. Finally, a third step was used to generate overlays involving the 1ndv ligand. This produced solutions close to the true overlay. Three other ordering strategies that seemed viable were also tried (detailed in Supporting Information). The results in Table 4 were obtained by ranking the pooled solutions from the four separate stepwise experiments. The top-ranked solution is similar to the true overlay.

### Heat shock protein 90

**Fig. 14:**
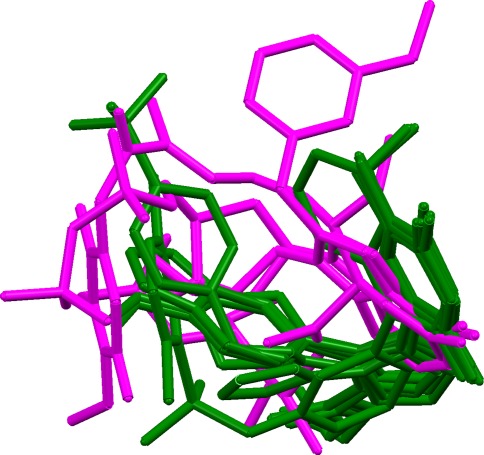
True overlay of ten heat shock protein ligands, showing the poor volume overlap of three ligands (from PDB complexes 1byq, 1uy8, 1yet, coloured in *magenta*) with the remainder (1yc1, 1yc4, 2bsm, 2byi, 2bz5, 2cct, 2uwd, coloured in *green*)

The 10 ligands in this set may be divided into two groups: (a) the closely similar pyrazole or isoxazole ligands of 1yc1, 1yc4, 2bsm, 2byi, 2bz5, 2cct, 2uwd together with the structurally unrelated ligand from 2bz5; and (b) two purine ligands (1byq and 1uy8) and the macrocyclic ligand from 1yet. All the ligands donate to Asp93 and accept from Thr184 and/or a conserved water molecule. However, the donor (D) and acceptor (A) groups in the group b ligands have shorter D···A distances than in the ligands of group a, potentially making the results sensitive to the choice of triplet distance bins. More seriously, the mouth of the binding cavity is rather large and the ligands from the two groups occupy different parts of it, resulting in poor volume overlap (Fig. 14). Consequently, reasonable results are obtained for subset HSP/1 (containing all the ligands in group a, but none from group b) but not for the complete set of all ten ligands, or for subsets such as HSP/2 which involve ligands from both groups. An additional problem with the HSP/2 subset is that many of the false solutions score better than the true overlay (for example, have far lower union volumes and better matching of hydrogen-bonding groups). Thus, it is not clear how the true overlay could be recognised even if it were generated. We conclude that this is a difficult test set.

### Acetylcholinesterase

**Fig. 15:**
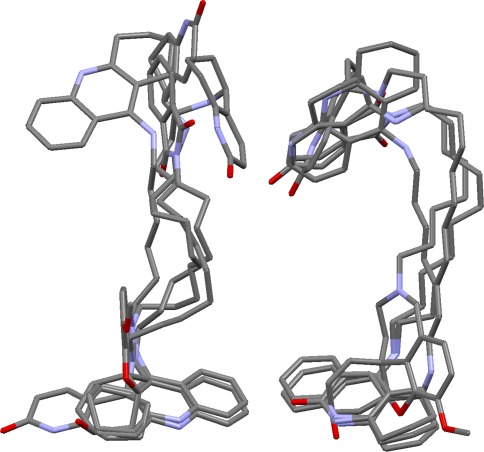
True overlay (*left*) and top-ranked prediction (*right*) for a subset of acetylcholinesterase ligands (1h23, 1w4l, 1zgb, 2ckm). The heterocyclic systems at the *top* are more closely aligned in the prediction than in the true overlay

This is the only protein on which we were entirely unsuccessful. No overlays at all (not even incorrect ones) can be generated for the complete set of eleven ligands because the algorithm cannot find any base triplet from which to construct an alignment fingerprint. The ligands from 2c5g and 1eve must be omitted (subset AChE/1) before any solutions can be produced, but nothing close to the true answer is found. The exceptional difficulty of the set may be explained as follows. (a) Binding is almost entirely hydrophobic in nature. No protein atom forms hydrogen bonds to more than three of the eleven ligands; several ligands form only one hydrogen bond to the protein; and one (1eve) forms no hydrogen bonds at all. Hydrophobic interactions are much less directional than hydrogen bonds, so this makes overlay prediction far harder. Further, most of the ligands have donor groups, so the algorithm tends to find false solutions with partial donor pharmacophore points. (b) The true overlay is in some respects less convincing than some of the solutions produced by the algorithm. For example, Fig. 15 shows the true and a predicted overlay for the subset of large ligands, AChE/2. In the true overlay, the heterocyclic systems in the vicinity of the AChE “peripheral binding site” (Trp279 and nearby residues) are much less closely overlaid that in the prediction. (c) There are significant ligand-induced conformational changes to the protein at Phe330, altering the space accessible to ligands.

Perhaps the biggest problem is that binding to AChE is dominated by electrostatic attraction between the electron-rich aromatic system of Trp84 and hydrophobic groups on the ligands that are rendered electron deficient by the inductive effect of nearby positive centres (all the ligands can safely be assumed to be cations). This type of electropositive hydrophobe is not specifically represented in our feature-typing scheme. Possibly, the set may be more amenable to field-based overlay methods [40].

### Influence of conformer input; computational requirements

Results obtained from the other conformer sets were inspected in sufficient detail to enable qualitative conclusions to be drawn. Optimising the geometry of the molecular models from which OMEGA generates conformers has variable effects. For DHFR, it is advantageous, since the geometry optimisation allows solutions to be found that have correct feature mappings and ligand conformations similar to those seen in the true overlay. In contrast, solutions generated from unoptimised starting points often have correct feature mappings but invariably with incorrect ligand conformations. For neuraminidase, however, optimisation was counter-productive, since it produced a poor geometry for the ligand from 1vcj, making it difficult to place correctly.

There seems to be a gradual improvement in results as the number of conformers is increased, but the effect is small. For example, the RAW200 set yields results for PK5, FABP, NEP, DHFR and Chk1 that are of are comparable quality to those obtained from the RAW5000 set, while the overlays for NEU are nearly as good. Computation times decrease dramatically for some sets as the number of conformers is reduced; example figures are given in Table 5. Memory requirements, which can be over a gigabyte in some cases, are also appreciably reduced by decreasing the number of input conformers. The faster speeds obtainable by using the smaller conformer sets probably outweigh any consequential loss in solution quality.

**Table 5:** Computation times (minutes) as a function of maximum number of conformers per ligand (based on RAW conformer sets)

	Maximum number of conformers per ligand		
Sets	5000	1000	200
NEP	38.4	19.8	4.2
DHFR	17.5	11.0	3.5
Chk1	2.8	2.6	1.8
NEU	3.6	3.6	3.4
CA	4.9	4.8	2.7
ADA	52.2	16.2	5.4

Elapsed times for overlay generation and filtering on an Intel T7500 2.2 GHz processor, excluding time required for conformer generation but including solution analysis

### Sensitivity of results to scoring functions

The hydrogen bond and hydrophobic scoring functions involve several empirical parameters and we wished to establish whether simpler functions might be adequate. Accordingly, we repeated the RAW5000 validation, replacing the hydrogen-bond score of Eq. (1) with the function:

7 $$ {\text{HB}}({\text{simple}}) = \Upsigma {\text{A}}_{\text{p}}^{2} $$

where the summation is over the clusters of donor atoms and acceptor atoms in the overlay, and A_p_ is the number of atoms in the pth such cluster. Similarly, the hydrophobic score was computed as:

8 $$ {\text{HY}}({\text{simple}}) = \Upsigma {\text{H}}_{\text{p}}^{2} $$

where the summation is over the clusters of hydrophobic groups in the overlay, and H_p_ is the number of hydrophobes in the pth such cluster. All other aspects of the methodology were unchanged. The results obtained were nearly as good as those described above (see Supporting Information), the only substantive difference being that the correct solution was no longer reliably obtained for ADA/1 using the stepwise method. We still prefer the more complicated functions because the score contributions made by individual clusters make more sense when these functions are used. For example, a buried cluster of hydrogen-bonding atoms in the true overlay of neuraminidase ligands gets a poor score contribution from Eq. (1) but a much larger contribution if (7) is used. The former is intuitively more reasonable and, in fact, the hydrogen-bonding atoms in question do not interact with the protein. Nevertheless, it is interesting that the simple functions perform so well.

## Conclusions

The alignment fingerprints described above have several useful characteristics. Each bit is set according to the presence or absence of a particular type of chemical feature at or near to a particular position in Cartesian space, when conformers are aligned in a consistent frame of reference defined by a triplet pharmacophore known to be present in all conformers contributing to the fingerprint. This is different from pharmacophore fingerprint techniques that assign bits according to the presence or absence of a pair of features separated by a particular distance [1]. The use of Cartesian-based fingerprints avoids the necessity of performing clique detection to confirm the presence of a pharmacophore, a step that is usually required when distance-based fingerprints are used. Every overlay generated from a fingerprint is guaranteed to have at least three full pharmacophore points, corresponding to the base triplet. The method takes into account partial as well as full pharmacophore points and is sensitive to whether two features in different molecules are exactly aligned (map to the same grid point) or only closely aligned (map to adjacent grid points). The B score calculated from the fingerprint correlates reasonably well with our more accurate hydrogen bond and volume scores, but is very quick to compute, allowing a large number of trial conformer combinations to be tested. The algorithm lends itself easily to constrained overlay generation.

In the validation, the algorithm performed well when the true overlay contained at least three full pharmacophore points. Thus, good results were obtained for the test sets PK5, FABP, NEP, DHFR, Chk1, NEU, ADA/2 and ADA/3: a high-ranking solution with the correct feature mappings and ligand conformations close to those seen in the PDB structures was almost always found. Results for CA (where the true overlay has the requisite 3 full pharmacophore points) were less good: the major features of the true overlay were predicted adequately but minor details were not reliably reproduced. For DHFR, solutions with correct feature mappings were easily found but tended to have incorrect ligand conformations. However, overlay multiplication was effective in finding alternative overlays with the same mappings but different conformations, including conformations similar to those in the crystal structures.

When the true overlay of a set of ligands did not contain three full pharmacophore points, the algorithm often performed poorly. This is sometimes understandable. For example, the binding of the 1krm ligand in the ADA set is very different from that of the other ligands. Similar situations occur in HSP with the 1byq, 1uy8 and 1yet ligands, and in AChE, where the binding of the 1eve ligand is idiosyncratic. Sometimes, false solutions look more convincing than the true overlay (for example, for the subsets HSP/2, AChE/2). If the true overlay looks unconvincing, it is likely to be overlooked by users even if it can be generated.

In the ADA/1 ligand set, however, the true overlay looks convincing even though it does not have three full pharmacophore points. Here, we were able to find the correct solution by a stepwise approach. This is an important proof of concept since it significantly extends the range of problems on which the algorithm might be successful (the use of fitting points on hydrophobe normals can also achieve this, since it can make a common pharmacophore of size 2 “look like” one of size 3). Currently, a limitation of the stepwise approach is that the user must choose manually the order in which the overlay is pieced together. This is probably not as hard as it sounds because it may often be obvious which ligands are causing difficulty, and the simple strategy of leaving these ligands to the end of the stepwise process may be successful, as it was for ADA/1. Nevertheless, an automated way of selecting the order in which ligands are introduced in stepwise overlay generation is an important goal for the future, as are a further investigation of the influence of the input conformer sets, validation against more test sets, and a systematic optimisation of program parameters.

## Electronic supplementary material

Below is the link to the electronic supplementary material.

Annotated ligand diagrams, tables listing the pharmacophore-point groups present in the true overlays of all test sets, definitions of hydrogen-bonding groups and rotatable-bond ranges, additional algorithm details, rmsds of best OMEGA approximations to binding-site conformations, details of steps used in ADA stepwise overlays, summary of results when simpler scoring functions are used. (DOCX 165 kb)
