# Supplementary material for: Development and validation of an improved algorithm for overlaying flexible molecules
Source: J Comput Aided Mol Des. 2012 Apr 27;26(4):451–72. doi: 10.1007/s10822-012-9573-y (PMC3348445; doi:10.1007/s10822-012-9573-y)
Supplement: Supplementary file 1 — Annotated ligand diagrams, tables listing the pharmacophore-point groups present in the true overlays of all test sets, definitions of hydrogen-bonding groups and rotatable-bond ranges, additional algorithm details, rmsds of best OMEGA approximations to binding-site conformations, details of steps used in ADA stepwise overlays, summary of results when simpler scoring functions are used. (DOCX 165 kb) [file 10822_2012_9573_MOESM1_ESM.docx]

**Supporting Information for “Development and Validation of an Improved Algorithm for Overlaying Flexible Molecules”**

Robin Taylor, Jason C. Cole, David A. Cosgrove, Eleanor J. Gardiner,

Valerie J. Gillet and Oliver Korb

**Contents**

1. Annotated ligand diagrams and tables of pharmacophore-point groups for all test sets
2. Definitions of hydrogen-bonding groups
3. Rotatable-bond range constraints
4. Additional algorithm details
5. Rmsds of best OMEGA approximations to binding conformations
6. Steps used in ADA stepwise overlays
7. Results of validation using simplified hydrogen-bond and hydrophobic scoring functions

PART A. ANNOTATED LIGAND DIAGRAMS AND TABLES OF PHARMACOPHORE-POINT GROUPS FOR ALL TEST SETS

For an explanation of the table column headings, see Table 3 and the “Success Criteria” section of the paper. Labels such as D1, A1, H1 in each chemical diagram indicate ligand features involved in interactions with the protein. Similar labels in the tables indicate which ligand features are involved in the pharmacophore-point groups. In the chemical diagrams, some ligand hydrogen-bond features may be given more than one label (e.g. both A1 and A2), indicating that they hydrogen bond in two or more directions to different protein atoms. Some ligand diagrams have feature labels that do not appear in the corresponding table (e.g. D1 in the 1tou diagram). This may indicate an atom or chemical group that forms an interaction to the protein that is formed by no other ligand in the set; or an interaction that is formed by two or more ligands in the set but was deemed to be relatively unimportant or ill defined, and was therefore not included in any pharmacophore-point group.

R_i_ values for pharmacophore-point groups in Table 4 of the paper are given in the same order as the pharmacophore-point groups are listed in the tables below, i.e. the nth R_i_ value given for a particular test set refers to the nth pharmacophore-point group in the table below corresponding to that test set.

**Pharmacophore-point groups for PK5 ligands.**

| group | description | full | partial | importance |
| --- | --- | --- | --- | --- |
| a | Donor and acceptor groups H-bonding to L82 (D1, A1); groups making hydrophobic contacts in vicinity of I10 (H1); groups making hydrophobic contacts in vicinity of L132 (H2); groups making hydrophobic contacts in vicinity of A30 (H3); groups making hydrophobic contacts in vicinity of V18, V63 (H4). | 6 | 0 | major |

**1v0o:**

**1v0p:**

**Pharmacophore-point groups for FABP ligands.**

| group | description | full | partial | importance |
| --- | --- | --- | --- | --- |
| a | Acid and hydroxyl groups accepting from R126, Y128 (A1, A2). | 1 | 1 | major |
| b | Groups making hydrophobic contacts in vicinity of F16, C117 (H2). | 1 | 0 | major |
| c | Groups making hydrophobic contacts in vicinity of M20, A75 (H1^*^). | 1 | 0 | major |
| d | Groups making hydrophobic contacts in vicinity of P38 (H1^*^). | 1 | 0 | major |

^*^ This large feature is divided into two parts.

**1tou:**

**1tow:**

**2hnx:**

**Pharmacophore-point groups for NEP ligands.**

| group | description | full | partial | importance |
| --- | --- | --- | --- | --- |
| a | Acidic groups (including thiolates) binding Zn, accepting from E584, H711 (A1, A2, A3). | 1 | 1 | major |
| b | Amide and imidazo groups donating to N542, accepting from R717 (D1, A4, A5). | 2 | 0 | major |
| c | Phenyl and isobutyl groups making hydrophobic contacts in vicinity of V580, H583 and W693 (H2). | 1 | 0 | major |
| d | Carboxylate oxygens accepting from N542 (A6). | 0 | 1 | moderate |

**1dmt:**

**1r1h:**

**1r1j:**

**1y8j:**

**Pharmacophore-point groups for DHFR ligands.**

| group | description | full | partial | importance |
| --- | --- | --- | --- | --- |
| a | Heterocyclic groups donating to E30 (D1, D4), I7 (D2), V115 (D3); and making hydrophobic interactions in vicinity of V8, A9 (H1) and L22, F34 (H3, H4). | 3 | 3 | major |
| b | Phenyl groups making hydrophobic contacts in vicinity of F31, F34, I60, L67 (H2, H5). | 0 | 2 | moderate |
| c | Amide carbonyls accepting from N64 (A2). | 0 | 1 | minor |
| d | Carboxylate oxygens accepting from R70 (A1, A3). | 0 | 2 | minor |

**1drf:**

**1hfr:**

**1mvt:**

**1pd9:**

**1s3v:**

**2dhf:**

**Pharmacophore-point groups for Chk1 ligands.**

| group | description | full | partial | importance |
| --- | --- | --- | --- | --- |
| a | Acceptor and donor groups (including some C-H donors) H-bonding to E85, C87 (A1, D1). | 2 | 0 | major |
| b | Groups making hydrophobic interactions in vicinity of G16, V23 (H2). | 0 | 1 | moderate |
| c | Groups making hydrophobic interactions in vicinity of L15, G90 (H3). | 0 | 1 | moderate |
| d | Donor groups H-bonding to C87 (D2). | 0 | 1 | minor |
| e | Groups making hydrophobic interactions in vicinity of K38, D148 (H4). | 0 | 1 | minor |

**1nvq:**

**1nvr:**

**1nvs:**

**1zlt:**

**1zys:**

**2br1:**

**2brb:**

**2brg:**

**2brh:**

**2brm:**

**2bro:**

**2c3l:**

**2cgu:**

**2cgw:**

**2cgx:**

**2hog:**

**Pharmacophore-point groups for NEU ligands.**

| group | description | full | partial | importance |
| --- | --- | --- | --- | --- |
| a | Acidic groups accepting from R116, R292, R374 (A1, A2, A3, A4); ring forming hydrophobic interactions in vicinity of Y409 (H1). | 3 | 0 | major |
| b | Amide or lactam carbonyl accepting from R150 (A5). | 1 | 0 | major |
| c | Groups forming hydrophobic interactions in vicinity of W177 (H2). | 0 | 1 | moderate |
| d | Guanidinium and bis(hydroxyl) side chains donating to E275 (D2, D3). | 0 | 2 | minor |
| e | Ammonium and hydroxyl substituents donating to E276 (D4). | 0 | 1 | minor |
| f | Guanidinium and hydroxyl groups donating to W177 (D6). | 0 | 1 | minor |

**1a4g:**

**1a4q:**

**1b9s:**

**1b9t:**

**1b9v:**

**1inf:**

**1inv:**

**1ivb:**

**1nsc:**

**1nsd:**

**1vcj:**

**Pharmacophore-point groups for CA ligands.**

| group | description | full | partial | importance |
| --- | --- | --- | --- | --- |
| a | Acidic groups coordinating metal atom and H-bonding to T199 (A1, A2, D1). | 2 | 0 | major |
| b | Rings (occasionally acyclic links) immediately adjacent to metal coordinators, forming hydrophobic interactions in vicinity of H94, V121, L198 (H1). | 1 | 0 | major |
| c | Sulfonamide, ether and thione groups accepting from Q92 (A3). | 0 | 1 | moderate |
| d | Groups forming hydrophobic interactions in vicinity of F131, P202 (H3). | 0 | 1 | moderate |
| e | Sulfonamide, ether and azole groups accepting from Q92 (A4). | 0 | 1 | minor |

**1bn3:**

**1bn4:**

**1bnq:**

**1cim:**

**1eou:**

**1if7:**

**1oq5:**

**1xpz:**

**1zgf:**

**1zh9:**

**2eu3:**

**2hoc:**

**2nng:**

**Pharmacophore-point groups for ADA ligands.**

| group | description | full | partial | importance |
| --- | --- | --- | --- | --- |
| a | Hydroxyl groups accepting from H17 and donating to D19 (A1, D3 [labelled as D6 in 1wxy] ). | 0 | 1 | moderate |
| b | Cationic donor groups salt-bridging to D295, D296 (D1, D2). | 0 | 2 | moderate |
| c | Groups forming hydrophobic interactions in vicinity of H17, F65 (H1). | 0 | 1 | moderate |
| d | Groups forming hydrophobic interactions in vicinity of L106, M155 (H2). | 0 | 1 | moderate |
| e | Groups forming hydrophobic interactions in vicinity of L62, G184 (H3). | 0 | 1 | moderate |
| f | Groups forming hydrophobic interactions in vicinity of F61, T269 (H4). | 0 | 1 | minor |

**1krm:**

**1ndv:**

**1ndw:**

**1ndy:**

**1o5r:**

**1qxl:**

**1uml:**

**1v7a:**

**1v79:**

**1wxy:**

**2e1w:**

**Pharmacophore-point groups for ADA/1 subset.**

| group | description | full | partial | importance |
| --- | --- | --- | --- | --- |
| a | Groups forming hydrophobic interactions in vicinity of L106, M155 (H2). | 1 | 0 | major |
| b | Hydroxyl groups accepting from H17 and donating to D19 (A1, D3 [labelled as D6 in 1wxy] ). | 0 | 1 | moderate |
| c | Cationic donor groups salt-bridging to D295, D296 (D1, D2). | 0 | 2 | moderate |
| d | Groups forming hydrophobic interactions in vicinity of H17, F65 (H1). | 0 | 1 | moderate |
| e | Groups forming hydrophobic interactions in vicinity of L62, G184 (H3). | 0 | 1 | moderate |
| f | Groups forming hydrophobic interactions in vicinity of F61, T269 (H4). | 0 | 1 | minor |

For ligand diagrams, see ADA above.

**Pharmacophore-point groups for ADA/2 subset.**

| group | description | full | partial | importance |
| --- | --- | --- | --- | --- |
| a | Hydroxyl groups accepting from H17 and donating to D19 (A1, D3 [labelled as D6 in 1wxy] ). | 1 | 0 | major |
| b | Groups forming hydrophobic interactions in vicinity of L106, M155 (H2). | 1 | 0 | major |
| c | Groups forming hydrophobic interactions in vicinity of L62, G184 (H3). | 1 | 0 | major |
| d | Groups forming hydrophobic interactions in vicinity of F61, T269 (H4). | 1 | 0 | major |
| e | Cationic donor groups salt-bridging to D295, D296 (D1, D2). | 0 | 2 | moderate |
| f | Groups forming hydrophobic interactions in vicinity of H17, F65 (H1). | 0 | 1 | moderate |

For ligand diagrams, see ADA above.

**Pharmacophore-point groups for ADA/3 subset.**

| group | description | full | partial | importance |
| --- | --- | --- | --- | --- |
| a | Cationic donor groups salt-bridging to D295, D296 (D1, D2). | 2 | 0 | major |
| b | Groups forming hydrophobic interactions in vicinity of L106, M155 (H2). | 1 | 0 | major |
| c | Groups forming hydrophobic interactions in vicinity of L62, G184 (H3). | 1 | 0 | major |
| d | Groups forming hydrophobic interactions in vicinity of F61, T269 (H4). | 1 | 0 | major |
| e | Hydroxyl groups accepting from H17 and donating to D19 (A1, D3). | 0 | 1 | moderate |
| f | Groups forming hydrophobic interactions in vicinity of H17, F65 (H1). | 0 | 1 | moderate |

For ligand diagrams, see ADA above.

**Pharmacophore-point groups for HSP ligands.**

| group | description | full | partial | importance |
| --- | --- | --- | --- | --- |
| a | Groups donating to D93 and accepting from conserved water or T184 (D1, A1). | 2 | 0 | major |
| b | Pyrazole NH groups donating to G97 (D3). | 0 | 1 | moderate |
| c | Phenolic OH groups donating to conserved water (D4). | 0 | 1 | moderate |
| d | Groups accepting from K58 (A6). | 0 | 1 | moderate |
| e | Groups making hydrophobic contacts in vicinity of M98, L107 (H4, 1yet excluded). | 0 | 1 | moderate |
| f | Groups making hydrophobic contacts in vicinity of F138, T184 (H5, 1yet excluded). | 0 | 1 | moderate |
| g | Amide NH groups donating to G97 (D5). | 0 | 1 | minor |
| h | Groups accepting from F138 (A5). | 0 | 1 | minor |

**1byq:**

**1uy8:**

**1yc1:**

**1yc4:**

**1yet:**

**2bsm:**

**2byi:**

**2bz5:**

**2cct:**

**2uwd:**

**Pharmacophore-point groups for HSP/1 subset.**

| group | description | full | partial | importance |
| --- | --- | --- | --- | --- |
| a | Groups donating to D93 and accepting from conserved water or T184 (D1, A1). | 2 | 0 | major |
| b | Groups making hydrophobic contacts in vicinity of M98, L107 (H4). | 1 | 0 | major |
| c | Groups making hydrophobic contacts in vicinity of F138, T184 (H5). | 1 | 0 | major |
| d | Pyrazole NH groups donating to G97 (D3). | 0 | 1 | moderate |
| e | Phenolic OH groups donating to conserved water (D4). | 0 | 1 | moderate |
| f | Groups accepting from K58 (A6). | 0 | 1 | moderate |
| g | Amide NH groups donating to G97 (D5). | 0 | 1 | minor |

For ligand diagrams, see HSP above.

**Pharmacophore-point groups for HSP/2 subset.**

| group | description | full | partial | importance |
| --- | --- | --- | --- | --- |
| a | Groups donating to D93 and accepting from conserved water or T184 (D1, D2, A1). | 2 | 0 | major |
| b | Groups making hydrophobic contacts in vicinity of M98, L107 (H4, 1yet excluded). | 1 | 0 | major |

For ligand diagrams, see HSP above.

**Pharmacophore-point groups for AChE ligands.**

| group | description | full | partial | importance |
| --- | --- | --- | --- | --- |
| a | Groups forming hydrophobic (cation-pi) interactions in vicinity of W84 (H2). | 1 | 0 | major |
| b | Groups forming hydrophobic interactions in vicinity of F330 (H3). | 0 | 1 | moderate |
| c | Groups forming hydrophobic interactions in vicinity of Y121, F331 (H5). | 0 | 1 | moderate |
| d | Groups forming hydrophobic interactions in vicinity of F330, Y334 (H6). | 0 | 1 | moderate |
| e | Groups forming hydrophobic interactions in vicinity of W279 (H9). | 0 | 1 | moderate |
| f | Pyridone carbonyl oxygens accepting from Y130 (A1). | 0 | 1 | minor |
| g | Anisole and phenolic oxygens accepting from water in oxyanion hole (A2). | 0 | 1 | minor |
| h | Pyridone NH groups donating to conserved water (D3). | 0 | 1 | minor |
| i | Cationic aromatic NH groups donating to H440 (D5). | 0 | 1 | minor |
| j | Hydroxyl and thiol groups donating to E199 (D6). | 0 | 1 | minor |

**1dx6:**

**1e66:**

**1eve:**

**1gpk:**

**1gpn:**

**1h23:**

**1w4l:**

**1zgb:**

**2ack:**

**2c5g:**

**2ckm:**

**Pharmacophore-point groups for AChE/1 subset.**

| group | description | full | partial | importance |
| --- | --- | --- | --- | --- |
| a | Groups forming hydrophobic (cation-pi) interactions in vicinity of W84 (H2). | 1 | 0 | major |
| b | Groups forming hydrophobic interactions in vicinity of F330 (H3). | 0 | 1 | moderate |
| c | Groups forming hydrophobic interactions in vicinity of Y121, F331 (H5). | 0 | 1 | moderate |
| d | Groups forming hydrophobic interactions in vicinity of F330, Y334 (H6). | 0 | 1 | moderate |
| e | Groups forming hydrophobic interactions in vicinity of W279 (H9). | 0 | 1 | moderate |
| f | Pyridone carbonyl oxygens accepting from Y130 (A1). | 0 | 1 | minor |
| g | Anisole and phenolic oxygens accepting from water in oxyanion hole (A2). | 0 | 1 | minor |
| h | Pyridone NH groups donating to conserved water (D3). | 0 | 1 | minor |
| i | Cationic aromatic NH groups donating to H440 (D5). | 0 | 1 | minor |

For ligand diagrams, see AChE above.

**Pharmacophore-point groups for AChE/2 subset.**

| group | description | full | partial | importance |
| --- | --- | --- | --- | --- |
| a | Groups forming hydrophobic (cation-pi) interactions in vicinity of W84 (H2). | 1 | 0 | major |
| d | Groups forming hydrophobic interactions in vicinity of F330, Y334 (H6). | 1 | 0 | major |
| e | Groups forming hydrophobic interactions in vicinity of W279 (H9). | 1 | 0 | major |

For ligand diagrams, see AChE above.

PART B. DEFINITIONS OF HYDROGEN BONDING GROUPS

Hydrogen-bonding groups are defined by the following SMARTS strings. The atoms in each group to be considered donors or acceptors are shown in bold. All charged groups are assigned hydrogen-bonding strength 3 (the highest strength). All others are of strength 2 except those followed by * in the list below, which are strength 1. Acceptors are classified as linear if the acceptor atom is sp hybridised, trigonal if sp^2^ hybridised, otherwise tetrahedral. This refers to the directions in which the acceptors prefer to form hydrogen bonds and controls placement of the virtual points used for computing hydrogen-bond scores (see text). If a group matches more than one donor SMARTS string, the first match (in the order shown below) is accepted; similarly for acceptors. If a group matches both a donor and an acceptor string, both are accepted. If the first acceptor SMARTS string matching a group is marked “IGNORE” in the list below, the atom shown in bold in that group is *not* considered an acceptor, even if it is part of a group matching a subsequent SMARTS string. This is a convenient way of ensuring that certain types of oxygen (e.g. in furan) are not treated as acceptors.

DONORS

[**N**X4.!H0]

[**N**X3.!H0]C(=[**N**X3.!H0])[**N**X3.!H0]

[**n**X3.!H0]c([**n**X3.!H0])[**n**X3.!H0]

[**N**X3.!H0]C=[**N**X3.!H0]

[**n**X3.!H0]c[**n**X3.!H0]

[B,C,N,P,S]=[**N**X3.!H0]-[H,B,c,C,n,N,O,F,P,S,Cl,Br,I]

a[**n**X3.!H0]a

[O,S]=C[**N**X3.!H0]

a-[**N**X3.!H0]

[C,N,O,P,S]=[C,N,P,S]-[**N**X3.!H0]

[C,N]#C-[**N**X3.!H0]

[**N**X3;!H0]

[**N**X2.!H0]

[**O**X2.!H0]

[**S**X2.!H0] *

[NX3]=[**C**X3.!H0] *

[nX3]-**C**H *

[nX3][**c**X3.!H0] *

N=[**C**X3.!H0]N *

n[**c**X3.!H0]n *

[CX2]#[**C**X2.!H0] *

ACCEPTORS

[**O**X1][CX3]=[**O**X1]

[**o**X1]~[cX3]~[**o**X1]

[**O**X1][PX4](=**O**)[**O**X1]

[**o**X1][pX4](~**o**)~**o**

[**O**X1][PX4](=**O**)

[**o**X1]~[pX4](~**o**)

[**O**X1][SX4](=**O**)=**O**

[**o**X1]~[sX4](~**o**)~**o**

c-[**O**X1]

[CX4]-[**O**X1]

[CX3]-[**O**X1]

[C,c,N,n]=[C,c,N,n]-[**O**R]-[C,c,N,n]=[C,c,N,n] IGNORE

[c,n]**o**[c,n] IGNORE

[C,c,N,n]=[C,c,N,n]-[**O**R]-[c,n] IGNORE

[H,C,c,N]-**O**[CX3]=[O,S] IGNORE

c-[**O**]-[CH3] IGNORE

C=C-[**O**]-[CH3] IGNORE

[**O**X2]

[C,c]~[NX3](~[C,c])[**O**X1]

[C,c][NX4]([C,c])([C,c])[**O**X1]

[OX2][SX4]([OX2])(=**O**)(=**O**)

**O**=[SX4](=**O**)[NX2][H,B,c,C,n,N,O,F,P,S,Cl,Br,I]

**O**=[SX4](=**O**)[NX3]

[**O**X1]=S([C,c])[C,c]

[**O**X1]S([C,c])[C,c]

[**O**X1]

[CX4][**N**X3;H2]

[NX3][**N**X3;H2]

[CX4][**N**X3;H1][CX4]

[CX4][**N**X3;H1][NX3]

[NX3][**N**X3;H1][NX3]

[CX4][**N**X3;H1]O

[CX4][**N**X3;H1][SX2]

[**N**X3]([CX4])([CX4])[CX4]

[**N**X3]([CX4])([CX4])[NX3]

[**N**X3]([CX4])([NX3])[NX3]

[**N**X3]([CX4])([CX4])[OX2]

[**N**X3]([CX4])([CX4])[SX2]

[**N**X3]([CX4])([CX4])[F,Cl,Br,I]

[**n**X2]

[H,B,c,C,n,N,O,F,P,S,Cl,Br,I]=[**N**X2][H,B,c,C,n,N,O,F,P,S,Cl,Br,I]

[H,B,c,C,n,N,O,F,P,S,Cl,Br,I][**N**X2][H,B,c,C,n,N,O,F,P,S,Cl,Br,I]

[CX2]#[**N**X1]

c-[**S**X1]

[CX4]-[**S**X1]

[CX3]-[**S**X1]

[NX3]C(=**S**)[NX3] *

PART C. ROTATABLE-BOND RANGE CONSTRAINTS

By default, all single acyclic bonds (and no others) are considered freely rotatable during overlay refinement and multiplication, provided it is possible to pick a valid torsion angle (i.e. the group is not linear, such as R-CN). This behaviour is overridden if a bond matches any of the SMARTS strings below. In this event, the conformation of that bond (as measured by the torsion angle defined by the atoms shown in bold) is restricted to the range(s) shown (ranges in degrees). If no range is shown, the bond is deemed non-rotatable, i.e. will be fixed at its starting conformation. If a bond matches more than one SMARTS string, the first match is accepted. The number alongside each range is its weight. Weights are used to bias random changes to torsion angles during overlay multiplication or refinement. Thus, if a rotatable bond does not match any of the SMARTS strings below, a random change to the torsion angle of that bond is equally likely to generate any value in the range -180- to +180°. If the bond does match one of the SMARTS strings, bias is introduced into the random torsion-angle sampling, such that a value in a range with weight W_1_ is more likely to be chosen than a value in a range with weight W_2_ by a factor W_1_/W_2_ (but all values within a range remain equally probable).

Users are able to control whether input conformers (i.e. conformers used at the overlay generation stage) must respect the rotatable-bond constraints. By default, all input conformers are accepted, even if they violate a constraint.

[**H,B,c,C,n,N,O,F,Si,P,S,Cl,Br,I**][**O**X2]**C**(=[O,S])[**H,B,c,C,n,N,O,F,Si,P,S,Cl,Br,I**]

-180 to -175, wt = 1

+175 to +180, wt = 1

[**B,c,C,n,N,O,F,Si,P,S,Cl,Br,I**][**N**X3]([B,c,C,n,N,O,F,Si,P,S,Cl,Br,I])**C**(=[O])[**H,B,c,C,n,N,O,F,Si,P,S,Cl,Br,I**]

-180 to -150, wt = 5

-30 to +30, wt = 10

+150 to +180, wt = 5

[**H,B,c,C,n,N,O,F,Si,P,S,Cl,Br,I**][**N**X3]**C**(=[O])[**H,B,c,C,n,N,O,F,Si,P,S,Cl,Br,I**]

-180 to -170, wt = 5

-10 to +10, wt = 1

+170 to +180. wt = 5

[**H,B,c,C,n,N,O,F,Si,P,S,Cl,Br,I**][**N**X3]**C(=[**S])[**H,B,c,C,n,N,O,F,Si,P,S,Cl,Br,I**]

-180 to -150, wt = 2.5

-30 to +30, wt = 1

+150 to +180, wt = 2.5

[**B,c,C,n,N,O,F,C,P,S,Cl,Br,I**]~[**B,c,C,n,N,O,C,P,S**][**C**H3]**H**

No ranges (fixed)

[**B,c,C,n,N,O,F,C,P,S,Cl,Br,I**]~[**B,c,C,n,N,O,C,P,S**][**N**H3]**H**

No ranges (fixed)

[**B,c,C,n,N,O,F,C,P,S,Cl,Br,I**]~[**B,c,C,n,N,O,C,P,S**][**N**X3H2]**H**

No ranges (fixed)

[**B,c,C,n,N,O,F,C,P,S,Cl,Br,I**]~[**B,c,C,n,N,O,C,P,S**][**N**X2H]**H**

No ranges (fixed)

[**B,c,C,n,N,O,F,C,P,S,Cl,Br,I**]~[**B,c,C,n,N,O,C,P,S**][**O**H]**H**

No ranges (fixed)

[**B,c,C,n,N,O,F,C,P,S,Cl,Br,I**]~[**B,c,C,n,N,O,C,P,S**][**S**H]**H**

No ranges (fixed)

[**B,c,C,n,N,O,F,C,P,S,Cl,Br,I**]~[**B,c,C,n,N,O,C,P,S**]**C**(**F**)(F)F

No ranges (fixed)

[**c,n**]**c**-**N**([**C**H3])[CH3]

No ranges (fixed)

[**C,N**]=**C**([C,N])-**N**(**H**)H

No ranges (fixed)

[**C,N**]=**C**([C,N])-**N**([**C**H3])[CH3]

No ranges (fixed)

PART D. ADDITIONAL ALGORITHM DETAILS

Volume score

All van der Waals radii are taken from Bondi A (1964) van der Waals Volumes and Radii. J Phys Chem 68:441-451. All atoms (including hydrogens) are included in the volume calculation.

Hydrogen-bond score

One, five and twelve virtual points, respectively, are placed to represent possible hydrogen-bonding directions for linear (sp), trigonal (sp^2^) and tetrahedral (sp^3^) acceptors (see text and Figure 1). For “hydrogen rotor” donors (–OH, -SH, –NH_3_^+^, pyramidal ‑NH_2_), virtual points are placed in an analogous manner to those of tetrahedral acceptors, i.e. twelve points equally spaced around the base of a cone. For all other donors, one point is placed on the extension of each donor-hydrogen vector. Virtual points are by default placed 3Å from the relevant donor or acceptor atom. When constructing nearest neighbour lists for the purposes of clustering virtual points, the same threshold distance is used as in the clustering of hydrogen-bonding atoms, i.e. 1.5Å.

In measuring steric accessibility, points are placed on the line between the centroid of the clustered donor or acceptor atoms and the centroid of the chosen virtual-point cluster, at 2.1, 2.4, 2.7 and 3.0Å from the former. Each point is examined to determine whether it falls within the hydrophobic envelope of the overlay, defined as being within a non-hydrogen atom that is not a donor or acceptor atom. The occlusion factor, X, varies from 1 if all four points lie outside the hydrophobic envelope to 0.1 if none of them do.

The similarity factor S is set to (m/n)^2^, where n is the actual number of hydrogen-bonding atoms in the cluster and m is an “effective” number. If all atoms in the cluster have the same strength, m is set to n. If there is a mixture of strong and medium (but no weak) or of medium and weak (but no strong), m is set to (2n-1)/2. If there are both strong and weak atoms in the cluster, m is first set to n–n(min), where n(min) is the minimum of n(strong) and n(weak), and then, if the cluster contains medium atoms, further reduced to (2m-1)/2.

By default, if two clusters, one of donors and one of acceptors, have at least 75% of their atoms in common (these must therefore be donor-acceptor atoms, typically hydroxyl oxygens), the less-highly scoring of the clusters is ignored.

Hydrophobic Score

The leader clustering is performed on the centroids of the directional hydrophobic groups but is otherwise exactly analogous to the procedure used for clustering hydrogen-bond donors and acceptors.

Customised Feature Score

The leader clustering is performed on the centroids of the customised features but is otherwise exactly analogous to the procedure used for clustering hydrogen-bond donors and acceptors.

Overlay Refinement

By default in the simulated annealing, 10000 moves are performed, the initial temperature is set to 0.05 times the score of the starting overlay, and is reduced by a factor of 0.95 every 250 moves. Each move involves a random change to a ligand torsion angle, or a rigid-body random translation or rotation of a ligand. The nature of each move (torsion, translation, or rotation) is chosen at random but with relative probabilities 6 : 1 : 1. Torsion changes are applied with equal probability to any of the rotatable bonds in the ligands but see Part C for a description of how torsion changes are affected by rotatable-bond range constraints. In a translation move, the maximum allowed translation is 0.05Å. In a rotation move, the maximum allowed Euler rotation is 0.2°.

We note that we are dissatisfied with the speed of simulated annealing and intend to move to a different refinement technique (see text).

Overlay Multiplication

The details of torsion, rigid-body translation and rigid-body rotation mutations are as for overlay refinement (see preceding section and Part C). The nature of each genetic operation (torsion mutation, torsion crossover, translation or rotation) is chosen randomly but with relative probabilities 6 : 2 : 1 : 1.

Tournament selection, for picking parents, is performed by default with a tournament group size of 3.

By default, populations can be distributed over a maximum of 50 niches, each able to accommodate a maximum of 3 chromosomes, and any two chromosomes (i.e. overlays) must be assigned to the same niche if they have a dissimilarity < 0.75, where dissimilarity is measured by the mean absolute difference between corresponding elements of the overlays’ inter-atom squared-distance matrices. For speed, these distance matrices are based on only a subset of the atoms of the ligands. This subset normally includes one atom from every feature of every ligand, viz. the atom closest to the centroid of each hydrophobic feature (based on the geometry of the first conformer provided by the user for each ligand), and all donor and acceptor atoms. However, it is advantageous to avoid topologically symmetric atoms so rather than selecting, for example, both oxygen atoms of a carboxylate group, the carboxylate carbon will be chosen instead. If the selection procedure ends up by choosing three consecutive atoms in a ligand (i.e. a bonded triplet A-B-C), the central atom will be dropped from the subset.

Each new generation is constructed by merging the parent and child populations, Pareto ranking them, and selecting in Pareto rank order, but rejecting any solution which would occupy a niche that has already been filled. In the case of a tie on Pareto rank, the older solution (based on the generation in which it was created) is chosen.

PART E. RMSDS OF BEST OMEGA APPROXIMATIONS TO BINDING CONFORMATIONS

Rmsds (Å) from least-squares superposition (allowing for topological symmetry) of non-hydrogen atoms of observed binding conformation on closest OMEGA conformer

| Protein | Ligand | RAW200 | OPT200 | RAW1000 | OPT1000 | RAW5000 | OPT5000 |
| --- | --- | --- | --- | --- | --- | --- | --- |
| PK5 | 1v0o | 0.47 | 0.71 | 0.47 | 0.71 | 0.47 | 0.71 |
| PK5 | 1v0p | 0.66 | 0.72 | 0.66 | 0.72 | 0.66 | 0.72 |
| FABP | 1tou | 0.72 | 0.71 | 0.70 | 0.62 | 0.70 | 0.62 |
| FABP | 1tow | 0.71 | 0.89 | 0.71 | 0.89 | 0.71 | 0.89 |
| FABP | 2hnx | 2.23 | 2.20 | 1.80 | 1.27 | 1.80 | 1.27 |
| NEP | 1dmt | 1.54 | 1.82 | 1.25 | 1.82 | 1.19 | 1.61 |
| NEP | 1r1h | 0.85 | 0.88 | 0.74 | 0.71 | 0.74 | 0.71 |
| NEP | 1r1j | 1.23 | 1.62 | 1.08 | 1.09 | 1.08 | 1.07 |
| NEP | 1y8j | 0.28 | 0.34 | 0.28 | 0.34 | 0.28 | 0.34 |
| DHFR | 1drf | 2.14 | 1.41 | 2.00 | 0.76 | 1.99 | 0.76 |
| DHFR | 1hfr | 0.92 | 1.35 | 0.87 | 1.17 | 0.87 | 1.17 |
| DHFR | 1mvt | 0.32 | 0.41 | 0.32 | 0.41 | 0.32 | 0.41 |
| DHFR | 1pd9 | 0.45 | 1.00 | 0.45 | 1.00 | 0.45 | 1.00 |
| DHFR | 1s3v | 0.20 | 0.87 | 0.20 | 0.87 | 0.20 | 0.87 |
| DHFR | 2dhf | 2.25 | 1.87 | 2.13 | 0.71 | 2.13 | 0.71 |
| Chk1 | 1nvq | 1.10 | 1.12 | 1.10 | 1.12 | 1.10 | 1.12 |
| Chk1 | 1nvr | 1.21 | 1.14 | 1.21 | 1.14 | 1.21 | 1.14 |
| Chk1 | 1nvs | 0.19 | 0.14 | 0.19 | 0.14 | 0.19 | 0.14 |
| Chk1 | 1zlt | 0.24 | 0.30 | 0.24 | 0.30 | 0.24 | 0.30 |
| Chk1 | 1zys | 0.38 | 0.36 | 0.38 | 0.36 | 0.38 | 0.36 |
| Chk1 | 2br1 | 0.47 | 0.49 | 0.47 | 0.49 | 0.47 | 0.49 |
| Chk1 | 2brb | 0.58 | 0.39 | 0.58 | 0.39 | 0.58 | 0.39 |
| Chk1 | 2brg | 0.73 | 0.54 | 0.73 | 0.54 | 0.73 | 0.54 |
| Chk1 | 2brh | 0.48 | 0.56 | 0.48 | 0.56 | 0.48 | 0.56 |
| Chk1 | 2brm | 0.46 | 0.59 | 0.46 | 0.59 | 0.46 | 0.59 |
| Chk1 | 2bro | 0.59 | 0.54 | 0.59 | 0.54 | 0.59 | 0.54 |
| Chk1 | 2c3l | 0.11 | 0.11 | 0.11 | 0.11 | 0.11 | 0.11 |
| Chk1 | 2cgu | 0.70 | 0.71 | 0.70 | 0.71 | 0.70 | 0.71 |
| Chk1 | 2cgw | 0.23 | 0.29 | 0.23 | 0.29 | 0.23 | 0.29 |
| Chk1 | 2cgx | 0.34 | 0.33 | 0.34 | 0.33 | 0.34 | 0.33 |
| Chk1 | 2hog | 0.50 | 0.53 | 0.50 | 0.53 | 0.50 | 0.26 |
| NEU | 1a4g | 0.66 | 0.63 | 0.66 | 0.63 | 0.66 | 0.63 |
| NEU | 1a4q | 0.75 | 0.59 | 0.75 | 0.59 | 0.75 | 0.59 |
| NEU | 1b9s | 0.79 | 1.06 | 0.79 | 1.06 | 0.79 | 1.06 |
| NEU | 1b9t | 0.45 | 0.55 | 0.45 | 0.55 | 0.45 | 0.55 |
| NEU | 1b9v | 0.37 | 0.50 | 0.37 | 0.50 | 0.37 | 0.50 |
| NEU | 1inf | 1.06 | 1.29 | 1.06 | 1.29 | 1.06 | 1.29 |
| NEU | 1inv | 0.30 | 0.84 | 0.30 | 0.84 | 0.30 | 0.84 |
| NEU | 1ivb | 0.94 | 0.95 | 0.94 | 0.95 | 0.94 | 0.95 |
| NEU | 1nsc | 0.83 | 0.86 | 0.83 | 0.86 | 0.83 | 0.86 |
| NEU | 1nsd | 0.30 | 0.29 | 0.30 | 0.29 | 0.30 | 0.29 |
| NEU | 1vcj | 0.45 | 0.98 | 0.45 | 0.98 | 0.45 | 0.98 |
| CA | 1bn3 | 0.72 | 0.75 | 0.72 | 0.75 | 0.72 | 0.75 |
| CA | 1bn4 | 0.72 | 0.66 | 0.50 | 0.66 | 0.50 | 0.66 |
| CA | 1bnq | 0.50 | 0.64 | 0.50 | 0.64 | 0.50 | 0.64 |
| CA | 1cim | 0.36 | 0.39 | 0.36 | 0.39 | 0.36 | 0.39 |
| CA | 1eou | 0.41 | 0.25 | 0.41 | 0.25 | 0.41 | 0.25 |
| CA | 1if7 | 0.78 | 0.76 | 0.51 | 0.45 | 0.51 | 0.45 |
| CA | 1oq5 | 0.63 | 0.64 | 0.63 | 0.64 | 0.63 | 0.64 |
| CA | 1xpz | 0.48 | 0.31 | 0.48 | 0.31 | 0.48 | 0.31 |
| CA | 1zgf | 0.24 | 0.25 | 0.24 | 0.25 | 0.24 | 0.25 |
| CA | 1zh9 | 0.68 | 0.31 | 0.50 | 0.31 | 0.50 | 0.31 |
| CA | 2eu3 | 0.19 | 0.29 | 0.19 | 0.29 | 0.19 | 0.29 |
| CA | 2hoc | 0.75 | 0.79 | 0.75 | 0.79 | 0.75 | 0.79 |
| CA | 2nng | 0.43 | 0.41 | 0.43 | 0.41 | 0.43 | 0.41 |
| ADA | 1krm | 0.50 | 0.43 | 0.50 | 0.43 | 0.50 | 0.43 |
| ADA | 1ndv | 0.39 | 0.44 | 0.39 | 0.44 | 0.39 | 0.44 |
| ADA | 1ndw | 0.27 | 0.29 | 0.27 | 0.29 | 0.27 | 0.29 |
| ADA | 1ndy | 0.43 | 0.55 | 0.43 | 0.55 | 0.43 | 0.55 |
| ADA | 1o5r | 0.83 | 0.83 | 0.83 | 0.83 | 0.83 | 0.83 |
| ADA | 1qxl | 1.25 | 1.11 | 0.98 | 0.99 | 0.98 | 0.99 |
| ADA | 1uml | 1.06 | 1.07 | 0.86 | 1.04 | 0.86 | 1.01 |
| ADA | 1v7a | 1.36 | 1.16 | 0.70 | 1.02 | 0.70 | 1.02 |
| ADA | 1v79 | 0.47 | 0.29 | 0.47 | 0.29 | 0.47 | 0.29 |
| ADA | 1wxy | 0.53 | 0.87 | 0.53 | 0.87 | 0.53 | 0.87 |
| ADA | 2e1w | 0.36 | 0.31 | 0.36 | 0.31 | 0.36 | 0.31 |
| HSP | 1byq | 1.09 | 1.03 | 0.88 | 0.94 | 0.88 | 0.85 |
| HSP | 1uy8 | 0.49 | 0.52 | 0.49 | 0.52 | 0.49 | 0.52 |
| HSP | 1yc1 | 0.40 | 0.52 | 0.40 | 0.52 | 0.40 | 0.52 |
| HSP | 1yc4 | 0.29 | 0.22 | 0.29 | 0.22 | 0.29 | 0.22 |
| HSP | 1yet | 2.88 | 2.88 | 2.88 | 2.88 | 2.88 | 2.88 |
| HSP | 2bsm | 0.36 | 0.43 | 0.36 | 0.43 | 0.36 | 0.43 |
| HSP | 2byi | 0.72 | 0.73 | 0.66 | 0.71 | 0.51 | 0.71 |
| HSP | 2bz5 | 0.44 | 0.57 | 0.44 | 0.57 | 0.44 | 0.57 |
| HSP | 2cct | 0.30 | 0.29 | 0.30 | 0.29 | 0.30 | 0.29 |
| HSP | 2uwd | 0.21 | 0.37 | 0.21 | 0.37 | 0.21 | 0.37 |
| AChE | 1dx6 | 0.18 | 0.16 | 0.18 | 0.16 | 0.18 | 0.16 |
| AChE | 1e66 | 0.40 | 0.37 | 0.40 | 0.37 | 0.40 | 0.37 |
| AChE | 1eve | 0.83 | 0.44 | 0.83 | 0.44 | 0.83 | 0.44 |
| AChE | 1gpk | 0.33 | 0.18 | 0.33 | 0.18 | 0.33 | 0.18 |
| AChE | 1gpn | 0.26 | 0.11 | 0.26 | 0.11 | 0.26 | 0.11 |
| AChE | 1h23 | 2.17 | 1.96 | 1.98 | 1.48 | 1.98 | 1.27 |
| AChE | 1w4l | 1.68 | 1.30 | 1.68 | 0.94 | 1.68 | 0.94 |
| AChE | 1zgb | 1.80 | 1.88 | 1.41 | 1.52 | 1.41 | 1.39 |
| AChE | 2ack | 0.31 | 0.30 | 0.31 | 0.30 | 0.31 | 0.30 |
| AChE | 2c5g | 0.08 | 0.10 | 0.08 | 0.10 | 0.08 | 0.10 |
| AChE | 2ckm | 1.10 | 1.09 | 1.02 | 0.99 | 0.60 | 0.83 |

PART F. STEPS USED IN ADA STEPWISE OVERLAYS

Ligands and/or supermolecules overlaid at each step of four different stepwise overlays performed on adenosine deaminase ligands (see text)

| Order | Step 1 | Step 2 | Step 3 |
| --- | --- | --- | --- |
| 1 | 1ndw, 1ndy, 1o5r, 1qxl, 1uml, 1v7a, 1v79, 2e1w | *(1ndw, 1ndy, 1o5r, 1qxl, 1uml, 1v7a, 1v79, 2e1w)*, 1wxy | *(1ndw, 1ndy, 1o5r, 1qxl, 1uml, 1v7a, 1v79, 1wxy, 2e1w),* 1ndv |
| 2 | 1ndw, 1ndy, 1o5r, 1qxl, 1uml, 1v7a, 1v79, 2e1w | *(1ndw, 1ndy, 1o5r, 1qxl, 1uml, 1v7a, 1v79, 2e1w)*, 1ndv | *(1ndv, 1ndw, 1ndy, 1o5r, 1qxl, 1uml, 1v7a, 1v79, 2e1w)*, 1wxy |
| 3 | 1o5r, 1qxl, 1uml, 1wxy | *(1o5r, 1qxl, 1uml, 1wxy)*, 1ndv | *(1ndv, 1o5r, 1qxl, 1uml, 1wxy)*, 1v7a, 1v79, 1ndw, 1ndy, 2e1w |
| 4 | 1ndv, 1o5r, 1qxl, 1uml | *(1ndv, 1o5r, 1qxl, 1uml*), 1wxy | *(1ndv, 1o5r, 1qxl, 1uml, 1wxy)*, 1v7a, 1v79, 1ndw, 1ndy, 2e1w |

*(x, y, z)* indicates supermolecules of ligands x, y, z, i.e. overlays of these ligands generated in the previous step

PART G. RESULTS OF VALIDATION USING SIMPLIFIED HYDROGEN-BOND AND HYDROPHOBIC SCORING FUNCTIONS

Results obtained from RAW5000 conformers^a^

| Set or subset | R_i_  (major) | R_i_  (moderate) | R_i_  (minor) | R_total_ | Seriously  misplaced | Rank, best | Rank, highest |
| --- | --- | --- | --- | --- | --- | --- | --- |
| PK5 | 0.5 | - | - | 0.5 | 0 | 2 | 2 |
| FABP | 0.3, 0.6, 0.4, 1.9 | - | - | 1.5 | 0 | 5 | 2 |
| NEP | 1.4, 0.4, 0.3 | 0.3 | - | 1.1 | 0 | 17 | 1 |
| DHFR | 0.4 | 1.3 | 0.2, 0.2 | 2.0 | 0 | 1 | 1 |
| Chk1 | 0.5 | 1.3, 1.0 | 0.6, 2.0 | 1.2 | 0 | 1 | 1 |
| NEU | 0.7, 0.3 | 0.4 | 0.7, 0.6, 0.9 | 0.7 | 1 | 1 | 1 |
| CA | 0.7, 0.7 | 1.0, 1.9 | 1.7 | 1.4 | 3 | 13 | 13 |
| ADA/1^b^ | - | 0.7, 0.6, 0.3, 1.0, 1.0 | 1.5 | 1.4 | 0 | 18 | 18 |
| ADA/2 | 0.8, 0.7, 0.5, 0.7 | 0.2, 0.2 | - | 1.7 | 0 | 4 | 1 |
| ADA/3 | 0.6, 0.6, 0.4, 1.9 | 0.1, 0.2 | - | 2.2 | 0 | 12 | 3 |
| HSP/1 | 0.5, 0.8, 0.2 | 0.2, 0.2, 0.9 | 0.1 | 0.8 | 0 | 11 | 7 |

^a^No satisfactory solutions were obtained for ADA, HSP, HSP/2; AChE, AChE/1, AChE/2

^b^Obtained by stepwise method
